# Mechanisms, detection, and impact of horizontal gene transfer in plant functional evolution

**DOI:** 10.1093/plcell/koaf195

**Published:** 2025-08-20

**Authors:** Lee Mariault, Camille Puginier, Jean Keller, Moaine El Baidouri, Pierre-Marc Delaux

**Affiliations:** Laboratoire Génome et Développement des Plantes, Université de Perpignan Via Domitia, 66860 Perpignan, France; Laboratoire Génome et Développement des Plantes, Centre National de la Recherche Scientifique, 66860 Perpignan, France; Laboratoire de Recherche en Sciences Végétales (LRSV), Université de Toulouse, CNRS, Toulouse INP, Castanet-Tolosan 31320, France; Laboratoire de Recherche en Sciences Végétales (LRSV), Université de Toulouse, CNRS, Toulouse INP, Castanet-Tolosan 31320, France; Laboratoire Génome et Développement des Plantes, Université de Perpignan Via Domitia, 66860 Perpignan, France; Laboratoire Génome et Développement des Plantes, Centre National de la Recherche Scientifique, 66860 Perpignan, France; Laboratoire de Recherche en Sciences Végétales (LRSV), Université de Toulouse, CNRS, Toulouse INP, Castanet-Tolosan 31320, France

## Abstract

Horizontal gene transfers (HGTs) have been observed across the tree of life. While their adaptive importance in bacteria is conspicuous, the occurrence of HGTs and their evolutionary significance in eukaryotes has only recently started to be considered. In this review, we explore the extent of HGT in the plant kingdom, indicating the widespread occurrence of microbe–plant HGT and plant–plant HGT. We propose mechanisms that mediate these transfers and detail the methods available to identify and test the robustness of putative HGT using both sequence-based and phylogenomic approaches. Exploring recently sequenced plant genomes across the green lineage has revealed hundreds of such HGTs. We discuss the impact of these transfers on plant adaptation and functional diversification. In the future, expanding the phylogenomic scrutinization of the plant kingdom should reveal the full extent of HGT. In situ sequencing and combinations of synthetic biology and experimental evolution may allow catching ongoing HGT and testing the functional relevance of such events.

## Introduction

Horizontal gene transfer (HGT)—the transfer of a piece of DNA from a donor organism to a recipient organism with mechanisms others than reproduction—is prevalent in bacteria, where it represents a common source of genetic novelty driving the emergence of functional innovations and adaptations (Arnold et al. 2022).

The occurrence of HGT in eukaryotes is far less common than in bacteria and varies between groups. For instance, protists and fungi are more prone to gaining genes by HGT than metazoan (Ocaña-Pallarès et al. 2022). Like in bacteria, HGT in eukaryotes is associated with trait evolution. Eukaryogenesis itself may be considered as a case of extreme HGT, with the primary endosymbiosis—the gain of a whole new genome by the host species—leading to the evolution of the first eukaryotic common ancestor. Another example of functional innovations associated with HGT is the acquisition of the nitrate assimilation pathway found in autotrophs and, among heterotrophs, in fungi and oomycetes, which originated in part from bacteria—eukaryote HGT, followed by multiple subsequent events of inter-eukaryote HGT (Ocaña-Pallarès et al. 2019).

As other eukaryotes, Viridiplantae also have been subjected to HGT. Among the most famous examples is the presence of transcriptionally active T-DNA from *Agrobacterium rhizogenes* in the genome of all tested cultivated sweet potatoes and their wild relatives (Kyndt et al. 2015; Quispe-Huamanquispe et al. 2019). Beyond this “naturally transgenic food crop” (Kyndt et al. 2015), the sequencing over the last decade of plant genomes covering the phylogenetic diversity of the lineage has started to reveal the extent of HGT in plants.

In this review, we discuss the potential mechanisms mediating HGT in plants. We describe existing methods to detect HGT in plant genomes and present examples illustrating the impact that ancient, and more recent, HGT have had on trait evolution in plants.

## Prevalence of HGT in plants genomes

Over the past few decades, an increasing number of studies have revealed that HGT is significantly more common in plant genomes than previously assumed. Plants not only acquire genes from other plant species but also engage in HGT with a diverse set of organisms across both prokaryotic and eukaryotic domains, including bacteria, fungi, insects, and even viruses. A comprehensive overview of documented HGT events in plants is provided in Table 1 and Supplementary Table S1.

**Table 1.** Representative horizontal gene transfer events in plants and their functional impacts

Authors	DOI	Transfer type	Donor species	Receiver species	Functional Impact
Hibdige et al.	10.1111/nph.17328	Plant-Plant	Multiple grass species	Multiple grass species	Enhanced adaptation and stress tolerance
Dunning et al.	10.1073/pnas.1810031116	Plant-Plant	Multiple grass lineages	*Alloteropsis semialata*	Stress responses, structural integrity, disease resistance, and senescence processes
Yang et al.	10.1038/s41477-019-0458-0	Plant-Plant	Various host species	Cuscuta campestris and related species	Contributing to metabolic capacity and parasitic ability
Yang et al.	10.1073/pnas.1608765113	Plant-Plant	Various host species	Striga, Phelipanche, Triphysaria, Phelipanche	Possible role in haustorium development
Prentice et al.	10.1098/rspb.2015.2453	Plant-Plant	A species of the grass genus Poa	*Festuca ovina*	Supports a role in fine-scale local adaptation
Fay-Wei Li et al.	10.1073/pnas.1319929111	Plant-Plant	Hornwort lineage	Fern lineage	Adaptation to low-light environments
Zhang, et al.	10.1186/1471-2229-14-19	Plant-Plant	*Arabidopsis thaliana* (Brassicaceae)	*Orobanche aegyptiaca and Cuscuta australis*	Potential role in alkaloid biosynthesis
Christin et al.	10.1016/j.cub.2012.01.054	Plant-Plant	C4 grass species	Alloteropsis species	Improved photosynthetic efficiency
Yoshida et al.	10.1126/science.1187145	Plant-Plant	Sorghum (Sorghum bicolor) or related grass species	Striga hermonthica	Likely involved in parasitism
Wang et al.	10.1038/s41477-025-01952-8	Plant-Prokaryote	Bacteria	Triticeae (wheat, barley, rye)	Enhanced drought tolerance, improved photosynthesis and increased yield
Puginier et al.	10.1038/s41467-024-48787-z	Plant-Prokaryote	Unclear	Trebouxiophyceae	Ability to lichenize
Marchant et al.	10.1038/s41477-022-01226-7	Plant-Prokaryote	Unclear	*Ceratopteris richardii*	Resistance to pathogens
Vancaester et al.	10.1093/molbev/msaa182	Plant-Prokaryote	Prokaryotes (various)	Diatoms	Expanded metabolic capabilities
Cheng et al.	10.1016/j.cell.2019.10.019	Plant-Prokaryote	Bacteria	*Spirogloea muscicola/Mesotaenium endlicherianum*	Various impacts (development, symbioses, responses to biotic/abiotic stresses…)
Fay-Wei Li et al.	10.1038/s41477-018-0188-8	Plant-Prokaryote	Bacteria	*Azolla*	Confers high insect resistance in ferns
Fang et al.	10.1038/s41598-017-05066-w	Plant-Prokaryote	Bacteria	Land Plants	Enhanced DNA repair mechanisms against UV radiation
Candotto-Carniel et al.	10.1007/s11103-016-0468-5	Plant-Prokaryote	Unclear	Trebouxiophyceae	Improvement of desiccation resistance
Yang et al.	10.1111/nph.13183	Plant-Prokaryote	Actinobacteria	Land Plants	Vascular development and terrestrial adaptation
Yue et al.	10.1038/ncomms2148	Plant-Prokaryote-Fungi-Virus	Prokaryotes, Fungi, Viruses	Early Land Plants (Moss)	Xylem formation, plant defense, nitrogen recycling as well as the biosynthesis of starch
El Mahboubi et al.	10.1101/2024.12.20.629586	Plant-Fungi	Unclear	Bryophytes	Antimicrobial
Beaulieu et al.	10.1038/s41588-024-02071-4	Plant-Fungi	Unclear	Embryophytes	Adaptation to biotic constraints
Liu et al.	10.1038/s41477-022-01129-7	Plant-Fungi	Unclear	*Cycas panzhihuaensis*	Insecticidal toxin
Watanabe et al.	10.1371/journal.pone.0257173	Plant-Fungi	*Epichloë* species	*Agrostis stolonifera* and related species	*AsBGNL*: pathogen resistance (soil-borne fungi); *AsFMOL*: pathogen defense/metabolism
Wang, et al.	10.1126/science.aba5435	Plant-Fungi	*Epichloë aotearoae*	*Thinopyrum elongatum* (wheatgrass)	Confers resistance to Fusarium head blight
Richards et al.	10.1105/tpc.109.065805	Plant-Fungi	Various fungi and plants	Non-angiosperms plants (Physcomitrella patens (2), lycophytes (3)) and fungi	Potentially aiding colonization of soil environments
Gilbert et al.	10.1093/gbe/evac141	Plant-Insect	Plant (unknown)	*Bemisia tabaci*	Nutrient provisioning and plant-interaction enzymes
Xia et al.	10.1016/j.cell.2021.02.014	Plant-insect	Plant (unknown)	*Bemisia tabac*i	Allows whiteflies to detoxify plant toxins
Lapadula et al.	10.1038/s41598-020-72267-1	Plant-Insect	Plant (unknown)	*Bemisia tabaci*, *Trialerodes vaporariorum*	Defensive or ecological advantages to whiteflies

**Figure 1.**
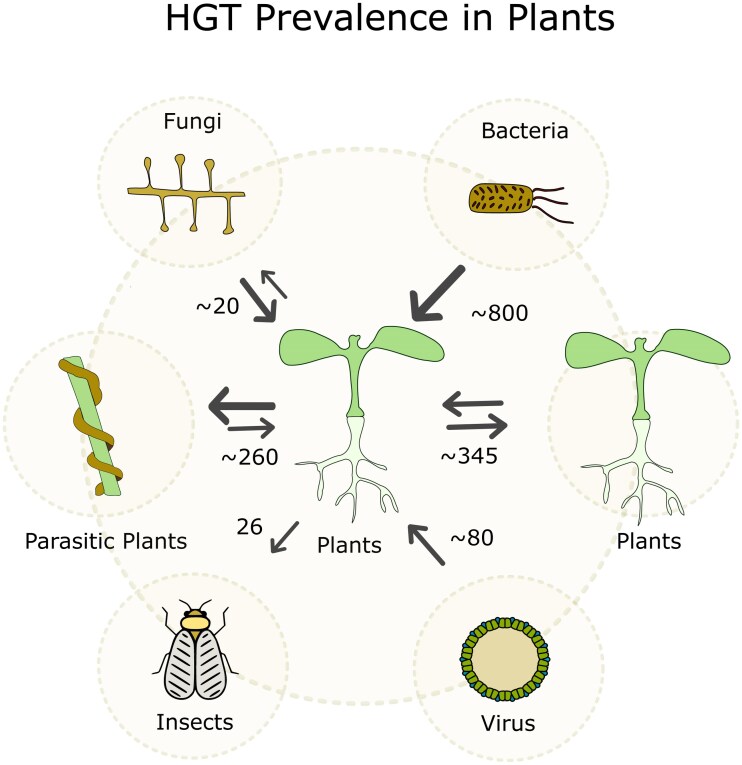
Prevalence of HGT in plants. Numbers and direction indicated are based on reported HGTs from the publications listed in Supplementary Table S1. Arrows start from donor lineage and point to the recipient lineage. A bigger arrow represents a higher frequency in a certain direction.

Plant-to-plant HGT is surprisingly widespread, with over 600 cases reported to date (Fig. 1). Notably, more than 42% of these involve few parasitic plants and their hosts. Members of the *Orobanchaceae* and *Convolvulaceae* families, in particular, have acquired hundreds of genes from a range of host plant species (Yang et al. 2016, 2019; Kado and Innan 2018). These extensive HGT events, which occur mainly from host to parasitic plants, are likely facilitated by the intimate and prolonged cell-to-cell contact through the haustorium, which is the organ that allows direct physiological and molecular exchange between parasitic plants and their hosts (see “*Mechanisms Mediating HGT*”). In contrast, HGT between non-parasitic plant species appears to be relatively rare, with the exception of HGT among related species within the *Poaceae* family (Dunning et al. 2019; Hibdige et al. 2021; Raimondeau et al. 2023). Remarkably, over 95% of the reported plant-to-plant HGT events outside of parasitic systems have occurred within this family. It remains unclear whether this apparent pattern reflects a true biological propensity for HGT among grasses or results from greater research focus and genomic data availability in this group.

HGTs are not limited to plant-to-plant events. Among all known sources, bacteria appear to be the most frequent donors of foreign genes to plant genomes (Fig. 1; Table 1). More than 800 genes have been reported to have been acquired from bacteria, particularly during key evolutionary periods such as the origin of land plants and the early divergence of seed plants (Yue et al. 2012; Ma et al. 2022). These HGTs have contributed to essential adaptive innovations HGT is not restricted to ancient events as more recent, lineage-specific transfers have also been identified. For instance, a recent study reported the acquisition of a bacterial gene by members of the *Triticeae* tribe, demonstrating that HGT continues to shape plant genomes in modern evolutionary time frames (Wang et al. 2025). Fungi are also recognized as donors of foreign genes in plants, although less frequently than bacteria. Approximately 20 well-supported fungal-to-plant HGT events have been described, some tracing back to early land plant evolution, while others appear to be more recent, particularly within the *Poaceae* family. Notably, a large proportion of these recent transfers involve endophytic fungi from the *Epichloë* genus, suggesting that intimate, long-term symbioses may facilitate the transfer of genetic material across kingdoms (Shinozuka et al. 2020; Wang et al. 2020a; Watanabe et al. 2021).

Although plant–insect HGT events appear to be rare, a remarkable case has been reported in the whitefly *Bemisia tabaci*, which acquired a plant-derived phenolic glucoside malonyltransferase gene representing a rare example of plant-to-animal HGT and highlighting unexpected direction HGT can take (Xia et al. 2021). More recently, additional cases of HGT from plants to *Bemisia tabaci* have been reported, with at least 25 plant-derived genes identified in the whitefly genome (Lapadula et al. 2020; Gilbert and Maumus 2022).

Finally, viruses—particularly those belonging to the *Caulimoviridae* family—represent another notable source of foreign genes in plant genomes. Viral sequences from this family have been found integrated into the nuclear genomes of a wide range of plant species, spanning both monocots and dicots (Geering et al. 2014).

## Mechanisms mediating HGT

**Figure 2.**
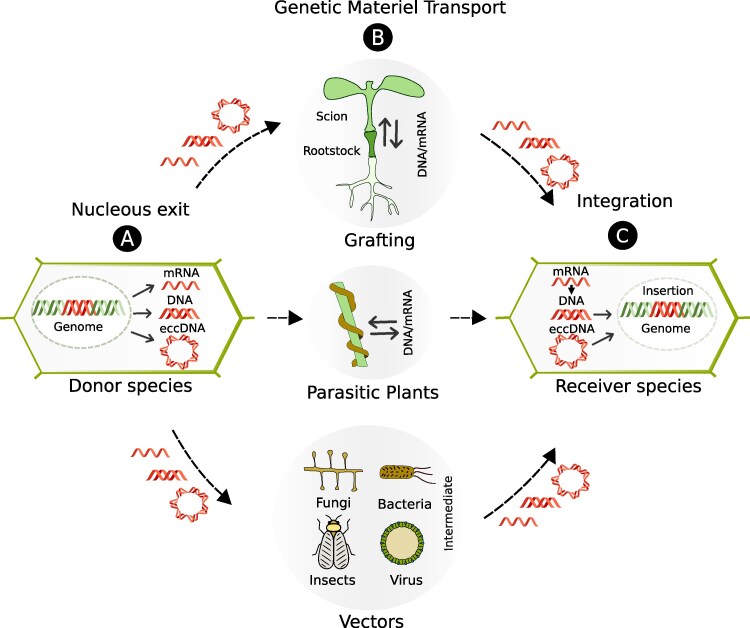
Putative mechanisms of HGT. (A) Nuclear exit. Genomic DNA, mRNA, or eccDNA exit the nucleus of the donor species' cell. (B) Genetic material transport. The genetic material is transferred to another species through vascular graft unions, parasitic haustorial organs, or vectors such as fungi, bacteria, insects, or viruses. (C) Integration of the genetic material. In the recipient species, foreign double-stranded DNA or eccDNA must enter the nucleus before integration into chromosomes. In the case of mRNA, reverse transcription is required prior to genomic integration. Solid arrows indicate intracellular movement; dashed arrows indicate interspecies transfer. Hexagons represent plant cells; circles labeled A–C mark the three stages.

Successful HGT requires the occurrence of several key steps (Fig. 2): 1) First, the genetic material must exit the nucleus of the donor cell into the cytoplasm; 2) the DNA must be transported to the recipient species cells, which may occur through direct cellular contact or via vectors (e.g. via viruses, endosymbionts, or other biological agents); and 3) the foreign DNA must reach the nucleus of the recipient cell and be integrated into the recipient genome for long-term persistence. Each of these steps presents physical and evolutionary challenges.

In this section, we will discuss the theoretical key steps required for HGT to occur in plants.

### How genetic material escapes the donor species cells

The initiation of a HGT event initially requires the export of genetic material from the nucleus to the cytoplasm. One of the main mechanisms facilitating this step is transcription, during which DNA is transcribed into mRNA, which is subsequently exported to the cytoplasm. This process generates a reservoir of genetic material that can serve as a substrate for HGT. Notably, mRNA molecules are highly mobile and can travel long distances between cells via plasmodesmata and the phloem (Luo et al. 2018; Kitagawa et al. 2024). This remarkable mobility makes mRNA a candidate for mediating HGT in plants. Indeed, there is evidence of frequent inter-specific mRNA exchange between parasitic plants and their hosts through specialized structures such as the haustorium (Kim et al. 2014).

Double-strand breaks caused by genotoxic stress could represent another pathway through which genetic material escapes the nucleus (Herbst et al. 2024). Apoptosis, which can occur in various plant tissues and at different developmental stages (Van Hautegem et al. 2015) may represent another source of small or large DNA fragments that might mediate HGT (Reape and McCabe 2008). However, experimental evidence demonstrating the stable presence of linear DNA in the cytoplasm remains elusive, as does evidence of their ability to cross plasmodesmata and inter-species barriers.

A further possibility is the involvement of extrachromosomal circular DNA (eccDNA) as a vector of HGT. Although a significant fraction of plant eccDNA is derived from transposable elements (TEs) (Zhang et al. 2023), functional genes have also been shown to form eccDNA (Peng et al. 2022). Specific sequences, such as ribosomal DNA and tRNA genes, are frequently detected in eccDNA, and larger genomic fragments up to 400 kbp containing genes can also form eccDNA (Koo et al. 2018). In some species, such as rice, eccDNA ranging from several dozens to 2 kbp long have been found to be enriched in coding sequences (Zhuang et al. 2024). The presence of eccDNA in plant cells, independently of chromosomes, raises the possibility that these molecules may facilitate HGT between plant species. Interestingly, a significant fraction of HT events identified in plants so far involve TEs, particularly LTR-retrotransposons (Baidouri et al. 2014; Aubin et al. 2023). The ability of LTR-retrotransposons to transfer frequently is likely due to their capacity to excise and mobilize as eccDNA during their life cycle (Lanciano et al. 2017). This may facilitate their movement between cells and potentially their “transferability” between species. This raises the intriguing possibility that gene-derived eccDNA, if capable of persisting in a stable from and migrating between cells, could mediate HGT in plants (Pereira and Dunning 2023).

### The touch that triggers horizontal gene transfer

#### Haustorium: the parasitic gateway

The occurrence of HGT depends on the ability of the genetic material of the donor species (DNA or mRNA) to physically reach the cells of the recipient species. Direct cell-to-cell contact, as seen in parasitic or endosymbiotic interactions, can facilitate this exchange across inter-species barriers. In parasitic plants, the haustorium forms a physical bridge between the parasite and its host, acting as a transmission route (Balios et al. 2025). This specialized organ allows the parasitic plant to connect to the xylem and/or phloem of the host, enabling the bidirectional movement of various molecules, including nucleic acids. Numerous cases of horizontal transfer of both nuclear and mitochondrial genes have been documented in parasitic plants belonging to *Orobanchaceae* (Yoshida et al. 2010; Zhang et al. 2013, 2014; Yang et al. 2016; Kado and Innan 2018), *Convolvulaceae* (Zhang et al. 2014; Yang et al. 2019), and various other parasitic plant families (Davis and Wurdack 2004; Mower et al. 2004; Sanchez-Puerta et al. 2017). These HGTs could be explained by the high frequency of bidirectional exchange of mRNA between parasitic plants and their hosts at the haustorial contact zone (David-Schwartz et al. 2008; Kim et al. 2014). Although direct evidence of systemic dispersal of mRNAs beyond the immediate haustorial contact zone remains limited (Thieme et al. 2015; Paajanen et al. 2025).

#### Mixing tissues by natural grafting

Natural grafting, the process of tissue fusion between 2 distinct plant species, is another potential route for HGT in nonparasitic plants facilitated by stock-scion mechanical contact. Experimental studies on transgenic tobacco lines have demonstrated that genetic material, including potentially entire plastids, can be transferred bidirectionally at the graft junction between different tobacco species (Stegemann and Bock 2009; Stegemann et al. 2012). Despite its potential role in facilitating HGT, the graft encounters obstacles, particularly the incompatibility and genetic divergence between species that can limit the efficiency of genetic exchange. While grafting between species within the same plant family is still possible, albeit with decreasing success, inter-familial grafting is always incompatible (Goldschmidt 2014). Consequently, grafting alone is unlikely to explain HGT between very divergent plant species, and the extent to which grafting contributes to HGT remains unclear.

#### Illegitimate pollination

Reproductive contamination through illegitimate pollination has been proposed as a potential mechanism facilitating HGT, particularly in wind-pollinated species such as grasses (Christin et al. 2012; Park et al. 2021; Pereira et al. 2023). This mechanism relies on the ability of heterospecific pollen to germinate (Lausser et al. 2010) and deliver exogenous DNA into the ovule, even if fertilization fails. Experimental evidence from repeated pollination and pollen tube pathway-mediated transformation suggests that foreign DNA can integrate into the recipient genome without the need for in vitro manipulation (Ali et al. 2015). While this mechanism is most plausible between closely related species where pollen tube growth is more likely to succeed, its contribution to HGT between more distantly related taxa remains uncertain.

#### Implication of vectors as a genetic bridge between donor and receiver species

One interesting hypothesis for explaining the transfer of genetic material between highly divergent plant species involves the mediation of an intermediate species from another kingdom, such as viruses, bacteria, nematodes, insects, or fungi. In this scenario, there is no direct cell-to-cell contact between the donor and recipient plant species. The occurrence of HGT between plant species with no known intimate cell-to-cell contact supports this hypothesis (Baidouri et al. 2014; Li et al. 2014; Mahelka et al. 2017; Dunning et al. 2019; Aubin et al. 2023). Additionally, there are multiple lines of evidence showing that plant genomes can capture by HGT genetic material from virus (Geering et al. 2014), bacteria (Yue et al. 2012; Kyndt et al. 2015; Li et al. 2018a; Cheng et al. 2019; Marchant et al. 2022; Puginier et al. 2024), and fungi (Shinozuka et al. 2020; Wang et al. 2020a; Watanabe et al. 2021; Li et al. 2022). Conversely, plant genes have also been transferred to various organisms including insects (Xia et al. 2021), fungi (Richards et al. 2009; Nikolaidis et al. 2014), and bacteria (Nikolaidis et al. 2014). An example of the implication of vectors in horizontal transfer in animals was previously reported. *Rhodnius prolixus*, a hematophagous parasitic insect, has acted as a genetic bridge by disseminating multiple transposon families among various vertebrate and invertebrate host species (Gilbert et al. 2010). Furthermore, viruses have been shown to encapsulate insect transposons that are inserted into their own genomes, underscoring their potential role as vectors for HT in animals (Gilbert et al. 2014). Despite these documented instances of cross-kingdom HT, no study to our knowledge has yet demonstrated the involvement of such vectors in plant-to-plant HGT. One plausible explanation for this gap is the inherent difficulty in detecting such events. Indeed, even if a vector transfers genetic material from a donor species to a recipient plant, it would be difficult to obtain direct evidence unless the transferred genes integrate into the vector's genome prior to transmission. Otherwise, no trace of the vector's involvement in HGT would remain. The growing availability of high-quality genome assemblies across diverse taxa will undoubtedly provide crucial insights into the potential role of vector in HGT.

#### Other possible routes

In the absence of direct evidence involving vectors, intimate cell-to-cell contact appears to be a key condition for HGT in plants. However, other types of interaction besides grafting (Stegemann and Bock 2009), parasitism (Davis and Wurdack 2004; Gilbert et al. 2010; Yoshida et al. 2010; Yang et al. 2019), and endosymbiosis (Shinozuka et al. 2020; Wang et al. 2020a; Watanabe et al. 2021) can also facilitate HGT. Close physical interactions between plants are widespread in natural ecosystems, offering virtually unlimited possibilities for contact between species. A study conducted in a forest ecosystem revealed frequent horizontal transfers between lianas and trees, as well as between different tree species (Aubin et al. 2023). Although these transfers involved only TEs, the possibility that genes could also be exchanged in the absence of clear cell-to-cell contact cannot be ruled out. It is also important to keep in mind that root systems, including rhizomes, continuously interact with soil microbiota, nematodes, and fungi potentially forming a highway for genetic material exchange between organisms. It is obvious that the transfer events identified so far are only the “tip of the iceberg,” and the biotic interactions facilitating HGT are probably much more abundant than is currently thought.

#### Touchdown of foreign genes in the recipient species genome

Once the genetic material reaches the recipient species, whether through direct cell-to-cell contact or via a vector, its journey is far from complete. Successful integration into the host genome is a prerequisite for stable inheritance. In cases where the transferred genetic material consists of mRNA, it must first undergo reverse transcription into complementary DNA before genomic integration. A well-documented example of this is the transfer of a gene of unknown function from parasitic plant *Striga* to its host sorghum (Yoshida et al. 2010). In sorghum, the transferred gene is intronless, in contrast to its homolog in *Striga*, providing indirect evidence that the transfer intermediate was an mRNA molecule. However, the exact mechanism by which this mRNA was reverse transcribed into DNA remains unknown, as does the question of whether reverse transcription occurred in the donor or the recipient species. Several reports have shown that plant genes could be reverse-transcribed into DNA and inserted back into the genome, leading to the formation of a retrogene (Panchy et al. 2016). However, the reverse transcription of mRNA likely requires the enzymatic activity of reverse transcriptases associated with TEs, such as Long Interspersed Nuclear Elements (Zhu et al. 2016) or LTR-retrotransposons (Tan et al. 2016). It is also plausible that certain viruses, such as those from the *Caulimoviridae* family (de Tomás and Vicient 2022), could contribute to the reverse transcription of host mRNAs, although direct evidence supporting this hypothesis is still lacking.

Most documented cases of HGT in plants do not appear to involve mRNA as an intermediary unlike the *Striga* example. This conclusion is supported by the presence of introns in the transferred genes in both the donor and recipient genomes, suggesting that the genetic material was transferred as DNA rather than reverse-transcribed mRNA (Yang et al. 2016, 2019). Overall, current evidence indicates that mRNA-mediated HGT in plants is rare. This scarcity may be attributed to the fact that reverse-transcribed mRNAs typically integrate without key regulatory elements, such as promoters and enhancers, preventing their proper expression. As a result, these sequences are prone to pseudogenization and eventual removal by purifying selection. Even when the transferred genetic material is in the form of linear or circular DNA, successful genomic integration requires nuclear import followed by stable incorporation into the receiver species' genome. Among the possible mechanisms facilitating the integration of foreign DNA, TEs may play a key role. In the coffee berry borer insect (*Hypothenemus hampei*), the acquisition by HGT of the *HhMAN1* gene (mannanase) from bacteria may have been facilitated by its insertion between 2 TEs belonging to the Tc1/mariner and hAT superfamilies (Acuña et al. 2012), suggesting a potential role of TEs in the integration of foreign genetic material. However, such a mechanism has not been demonstrated so far in plant models.

### Dead end or transgenerational horizontal gene transfer?

The successful integration of foreign genes into the cells of a recipient species is not a sufficient condition for transgenerational inheritance. If the integration of foreign genes occurs in vegetative tissues, it is unlikely that the transferred genes will be transmitted to the next generation, resulting in an evolutionary dead end. On the other hand, when HGT occurs in germ cells, there is a significant chance that the foreign DNA will be inherited by the offspring. In bryophytes, the gametophyte is the dominant phase of the life cycle and is more exposed to the environment, thereby increasing the likelihood of acquiring foreign genes through HGT and transmitting them to the next generation (Li et al. 2014). In contrast, most other land plants, particularly seed plants (spermatophytes), have highly reduced and protected gametophytes enclosed within sporophytic tissues, minimizing direct environmental exposure. Nonetheless, most documented cases of plant HGTs have been identified in spermatophytes, which suggests the existence of a pathway allowing foreign genes to become transgenerational in these species.

There are 2 main developmental and reproductive traits in plants that may influence the likelihood of transgenerational HGT: 1) First, unlike animals, where germ cells appear early during embryonic development, plant germ cells usually arise late from somatic (vegetative) cells and may be regenerated multiple times throughout the organism's lifespan. This developmental feature increases the probability that vegetative cells containing foreign DNA could eventually give rise to germ cells, thereby allowing the transmission of transferred genes to subsequent generations. 2) Second, some plant species can reproduce asexually, allowing the generation of entire individuals from vegetative tissues. Vegetative reproduction enables clonal propagation enhanced by stress conditions through various organs, including roots, stems, and leaves, which could allow transgenerational inheritance of somatic HGT (Anderson et al. 2001; Ott et al. 2019; Yu et al. 2020). In certain species roots develop adventitious buds, which can detach and grow into independent plants, as observed in *Siphanthera arenaria* (Menezes-e-Vasconcelos and Melo-de-Pinna 2024). Similarly, tuberous adventitious roots, such as those in *Ipomoea batatas* and *Dahlia*, not only store nutrients but also generate buds capable of developing into new individuals. In addition, many spermatophytes reproduce vegetatively via stem modifications, including rhizomes (e.g. *Musa paradisiaca*, *Zingiber officinale*), corms (*Amorphophallus*, *Colocasia*), tubers (*Solanum tuberosum*), bulbs (*Allium cepa*, *Lilium*), runners (*Centella asiatica*), stolons (*Mentha*, *Fragaria*), suckers (*Chrysanthemum*), and bulbils (*Dioscorea*, *Agave*). Moreover, some species, such as *Bryophyllum* and *Begonia*, propagate through epiphyllous buds formed on leaves, which develop into independent plants upon detachment (Dickinson 1978). These structures, which generate new plants through the activation of axillary or adventitious buds, provide a potential route for the persistence and inheritance of horizontally transferred genes. This suggests a non-negligible probability that foreign DNA integrated into the somatic cells of these vegetative structures could be maintained across generations, initially through clonal propagation and subsequently through sexual reproduction.

Notably, underground vegetative buds derived from roots or stems are in direct contact with the rhizosphere, which harbors a diverse community of organisms, including fungi, bacteria, nematodes, and insects. Many of these organisms engage in symbiotic or parasitic interactions, thereby increasing the likelihood of exposure to foreign DNA. Consequently, while the enclosure of gametophytes in seed plants minimizes direct environmental exposure, the widespread occurrence of vegetative reproduction presents an alternative and plausible route for transgenerational HGT in spermatophytes. However, it remains unclear whether vegetative reproduction in plants plays a significant role in facilitating successful transgenerational HGT.

## Methods and criteria for inferring HGT

Detecting HGTs remains a major challenge in evolutionary biology. The characterization of their genomic signatures relies on a straightforward principle: when a gene is transferred between 2 species, the sequences of the donor and recipient copies are initially identical. This is reflected not only in high sequence similarity but also in phylogenetic incongruence between the transferred gene and the species phylogeny, as well as patchy taxonomic distributions, among other signatures (Bock 2010). These various genomic signatures caused by HGTs serve as common detection criteria used to infer horizontal transfer events. Therefore, the robust identification of HGT events requires the integration of multiple lines of evidence and often necessitates both in silico and experimental validation to rule out contamination. The following section explores these criteria, discussing their respective strengths and potential limitations.

### Sequence-based criteria

#### High sequence similarity

**Figure 3.**
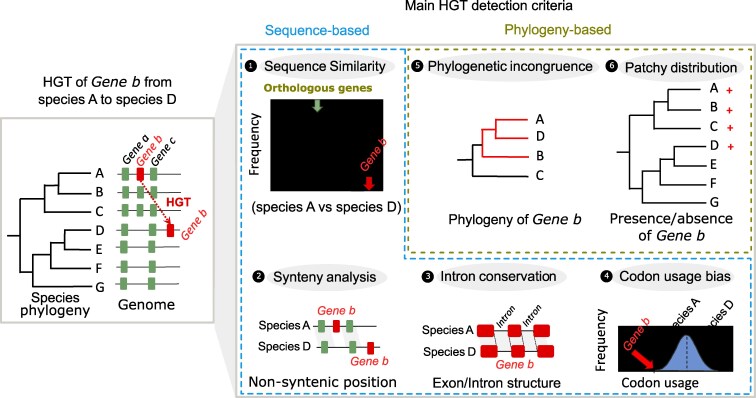
Approaches for detecting HGT. Left panel: a species tree illustrates HGT of Gene b from donor species A to recipient species D, contrasting with vertically inherited Genes a and c. Right panel: The 6 main HGT detection criteria are grouped into sequence-based and phylogeny-based evidence. Sequence criteria: (1) unusually high donor-recipient sequence identity; (2) Absence of synteny between donor and recipient species of the horizontally transferred genes following its integration in the recipient genome; (3) Conservation of both exon and intron sequences between highly divergent species is hallmark of HGT (4) Highly divergent codon usage of foreign gene compared with receiver genes and it's resemblance to donor codon usage. Phylogeny criteria: (5) gene-tree vs species-tree incongruence; (6) patchy presence/absence across taxa. The “+” symbol represents presence of homolog of Gene b.

When HGT occurs, the sequence of the transferred gene is very similar, often almost identical, between the donor species and the recipient species. As a result, when sequence similarity is assessed at the nucleotide or protein level, transferred genes generally exhibit much higher similarity than orthologous genes inherited vertically from a common ancestor (Fig. 3). This unexpectedly high sequence identity between genes from distantly related species serves strong indicator of HGT (Peccoud et al. 2018; Wickell and Li 2020; Aubin et al. 2021). However, its reliability depends on several key factors: the evolutionary distance between the species involved, the age of the transfer, and the selective pressures acting on the transferred gene. In the case of a recent HGT between distantly related species, elevated similarity is typically straightforward to detect, as the transferred gene is significantly more conserved than genes inherited through vertical transmission. Over time, however, and depending on selection pressure acting on the gene in donor and receiver species (whether positive, neutral, or negative), similarity may erode until it becomes indistinguishable from that of vertically inherited orthologs. Conversely, when HGT occurs between closely related species, the transferred gene may initially exhibit a high degree of similarity, but the difference from vertically inherited genes is generally less significant than in the case of more distantly related donor. Furthermore, in the absence of strong selective constraints, sequence similarity can decrease relatively quickly. Thus, the detectability of HGT based on sequence similarity is constrained within a “window of HGT observation,” a conceptual range defined by genetic divergence, the age of the transfer, and the fate of the gene in both donor and recipient lineages (e.g. retention, pseudogenization, or positive selection). This generally corresponds to the period during which sequence identity remains anomalously high relative to expectations under vertical inheritance. Beyond this window, distinguishing HGT from other evolutionary processes becomes increasingly ambiguous. Finally, another factor that could significantly impact this observation window is the availability and resolution of genomic data. In many cases, the true donor species is unknown, either because taxonomic sampling around the putative donor is limited or because the actual donor was an ancestral species that is now extinct. As a result, analyses often rely on a proxy species as the presumed donor. The genetic distance between this proxy and the true donor further increases the overall divergence, making the detection and interpretation of HGT events even more challenging.

#### Intron conservation

Intron similarity between the donor and recipient species provides a strong indication of HGT occurrence. While exons are often conserved due to purifying selection, introns typically evolve more rapidly and are rarely shared between distantly related species (Long et al. 1995; Laroche et al. 1997). Therefore, when a gene's introns in the recipient species closely match those of the donor, this strongly suggests HGT rather than vertical inheritance (Richardson and Palmer 2007) (Fig. 3). Unlike exonic sequences, which may be constrained by strong selection pressure, the presence of highly conserved and intronic regions in 2 unrelated species is unlikely the result of strong selection or convergent evolution (Xi et al. 2012). However, long-term divergence may obscure intron-based signals in older transfers, and some HGT events involve intronless genes or partial gene fragments, limiting this approach (Yoshida et al. 2010).

#### Codon usage bias

Codon usage bias provides another clue for detecting HGT, as it captures patterns of synonymous codon preferences that can vary significantly across species. For instance, dicots often use a broader range of codons ending in A or U, whereas monocots may exhibit narrower preferences favoring C- or G-ending codons (Murray et al. 1989; Campbell and Gowri 1990). A gene displaying a significant discrepancy between its codon usage and the typical pattern of the host plant may indicate a foreign origin through HGT (Won and Renner 2003; Friedman and Ely 2012). However, codon usage is influenced by factors such as translational selection and mutation biases (Wang et al. 2018; Chakraborty et al. 2020). Moreover, variations in codon usage are notable even within taxonomic groups, influenced by gene-specific factors such as expression level and selective pressures related to translational optimization or mRNA stability. Consequently, this criterion is often used as additional support for a suspected HGT rather than as a primary screening method.

#### Synteny analysis

The integration of a foreign gene into a recipient species places that gene in a new genomic context compared with the donor species, which can serve as evidence supporting HGT (Adato et al. 2015; Sevillya et al. 2020). Indeed, the probability that a foreign gene would integrate exactly into the same genomic position as in the donor genome is extremely low (Fig. 3). Although nonsyntenic conservation of homologous genes in plants can arise through various evolutionary processes (e.g. gene duplication, loss, or structural variation) and is therefore not exclusively a hallmark of HGT, horizontally transferred genes are invariably present in nonsyntenic position between donor and receiver genome. Conversely, if a gene exhibits multiple signatures indicative of HGT yet retains the same genomic context as its putative donor, this invalidates the HGT hypothesis.

### Phylogenetic approaches

#### Phylogenetic incongruence

Under strict vertical inheritance, a gene's phylogenetic tree should generally mirror the well-resolved species tree. A pronounced incongruence between the transferred gene phylogeny and species phylogeny is as strong evidence for HGT, particularly if the gene clusters with a taxon distantly related to the focal species (Hill et al. 2010) (Fig. 3). However, phylogenetic incongruences can also arise from various biological and technical artifacts. Processes such as gene duplication and subsequent loss, incomplete lineage sorting, and hybridization can all generate incongruent topologies (Stolzer et al. 2012).

Several complementary strategies can be used to detect and assess phylogenetic incongruence (Steenwyk et al. 2023). Statistical tests, such as the AU test (Shimodaira 2002), evaluate whether a gene tree significantly conflicts with the species tree. Measures of bipartition support, including concordance factors, gene support frequencies, and internode certainty, quantify how much of the data supports specific branches and highlight conflicting signals. Phylogenetic networks and split graphs provide a useful way to visualize alternative relationships that may not fit a strictly bifurcating tree. Reducing methodological artifacts can also help, for example by using partitions less influenced by selection, such as 4-fold degenerate sites or introns. Finally, probabilistic models that infer gene family evolution by reconciling duplications, transfers, and losses—as implemented in tools like SpeciesRax (Morel et al. 2022) and Ranger-DTL (Bansal et al. 2018)—offer a powerful framework for inferring gene transfer events and provide a more accurate explanation of phylogenetic incongruence.

#### Patchy distribution

A “patchy” taxonomic distribution occurs when a transferred gene is present in distantly related species but absent from their closest relatives (Fig. 3). Such discontinuous gene distribution can indicate HGT, particularly if there is no robust evidence supporting vertical gene loss in lineages lacking the gene. However, incomplete species sampling and low-quality genome assemblies can artificially create patchiness (Zhu et al. 2014). There are also cases where patchy distributions cannot be assessed, especially when homologs of the transferred gene are already present in recipient species. Consequently, clear patchy distribution patterns are most reliably confirmed between highly divergent lineages, where homologous genes are unlikely to have arisen through vertical inheritance alone.

#### Rejecting the contamination hypothesis

Contaminant sequences pose a critical challenge in HGT studies. This is especially true when HGT occur between intimately associated organisms such as between parasites or endophytes and their host plants. In these situations, genome sequencing could yield metagenomic mixtures rather than genomes from a single species. As a result, assembled plant genomes may harbor exogenous DNA from bacterial or fungal parasites and endophytes. To rigorously validate HGT events, it is important that the transferred sequences are embedded within large contigs or scaffolds and flanked by bona fide host sequences (Jiao et al. 2017; Belser et al. 2018; Michael and VanBuren 2020). For instance, in cases of HGT between a fungus and a plant, it is essential to demonstrate that the fungal-derived gene is flanked by plant-specific sequences in the assembly. This could be further supported by analyzing raw sequencing reads to confirm the physical anchoring of the transferred sequence within the recipient genome. When genome assembly was produced using long-read sequencing technologies (e.g. PacBio, Oxford Nanopore), it is crucial to confirm the presence of raw reads spanning the transferred sequence along with its surrounding genomic context. Additionally, experimental validation techniques, such as PCR amplification of the junction between the putative foreign and host-derived sequences followed by sequencing, provide direct evidence of the genomic integration of the transferred gene (Quispe-Huamanquispe et al. 2017; Aubin et al. 2023). However, the effectiveness of these validation approaches can be influenced by the age of the transfer, particularly in cases where the HGT event is very recent and still polymorphic within the population (Dunning et al. 2019).

## The great diversity of HGTs played a role in plants adaptation and functional evolution

**Figure 4.**
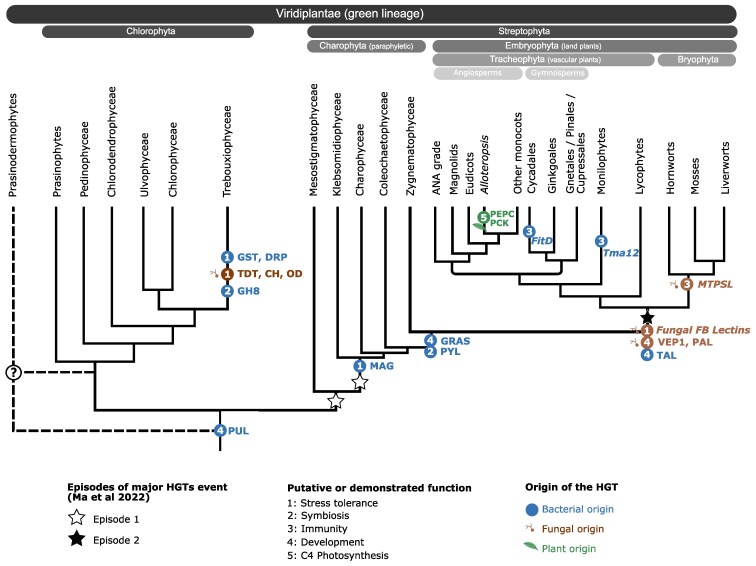
HGTs contributed functional evolution in plants. The 2 episodes of major HGT events as described in Ma et al. (2022) are indicated with white (episode 1) and black (episode 2) stars. Examples of genes originating from HGT that contributed innovations in the plant lineages are indicated on the plant phylogeny. Pullulanase (*PUL*), glutathione-s-transferase (*GST*), Desiccation-Related Proteins (*DRP*), Glycoside Hydrolase family 8 (*GH8*), Tellurite-resistance dicarboxylase (*TDT*), Nitrilase/cyanide hydratase (*CH*), Oxidoreductase/retinol dehydrogenase (*OD*), DNA-3-methyladenine glycosylase (*MAG*), insecticidal toxin (*FitD*), lytic polysaccharide monooxygenase (*Tma12*), Microbial Terpene Synthase-Like (*MTPSL*), Fungal Fruit Body Lectins (Fungal FB Lectins), Vein Patterning 1 (*VEP1*), Transaldolase type A (*TAL*), ortholog of the PYL abscisic acid receptors (*PYL*), GRAS transcription factors (*GRAS*), phenylalanine ammonia lyase (PAL), *PEPC* (PEP Carboxylase), *PCK* (PEP carboxykinase). The putative or demonstrated functions of horizontally acquired genes are numbered from 1 to 5.

The identification and analysis of HGTs discussed in the previous sections underscore the extensive diversity of these events, although certain lineages and functions have received more attention than others. Notably, a HGT event that may have played a central role in the evolution of the entire green lineage is the acquisition of a bacterial pullulanase (Fig. 4) (Yue et al. 2012; Qu et al. 2018), an enzyme involved in starch biosynthesis (Ma et al. 2018), illustrates how HGT can facilitate key metabolic innovations. More generally, (Ma et al. 2022) showed that 2 major HGT episodes shaped the evolution of streptophytes and embryophytes (Fig. 4) and were followed by more recent lineage specific innovations (Ma et al. 2022; Wang et al. 2023). For instance, the role of HGTs in the evolution of parasitic plants has been well documented (Davis and Xi 2015; Wickell and Li 2020; Aubin et al. 2021). Another prominent example is the contribution of HGTs to the emergence of C4 photosynthesis in grasses. This photosynthetic pathway enhances carbon fixation efficiency in certain plant lineages. Enzymes integral to the C4 cycle, such as PEP carboxylase and PEP carboxykinase, have been horizontally acquired in the genus Alloteropsis (Fig. 4), likely via illegitimate pollination (Christin et al. 2012). These findings support the view that HGTs can act as important drivers of functional innovation in C4 grasses. Beyond individual cases of metabolic innovation, HGTs have also supported major evolutionary transitions and processes involved in plant-microbe interactions, underscoring the broader significance of HGT in plant adaptation and diversification.

## HGT supported major transitions during plant evolution

Plants evolution is paved by the emergence of functional innovations at the origin of their astonishing diversification and adaptation to all biomes on Earth. These innovations have genetic bases and part of the genetic novelties derive from HGT. Among the most important transitions, the terrestrialization of plants, approximately 450MYA, stands out as it paved the road for other macro-organisms to adapt and diversify on land (Rensing 2018). The transition from water to land required the evolution of functional innovations to adapt to new constraints such as desiccation, UV radiation or limited availability of nutrients (Rensing 2018). Such innovations evolved through the acquisition of new genetic functions and part of them were horizontally acquired concomitantly with the terrestrialization process.

### Several HGTs correlate with the plant terrestrialization event

The significant increase in the production of genome assemblies covering the different land plant lineages enabled large-scale and genome-wide comparisons to identify HGTs putatively involved in the adaptation of plants to land. This led to the identification of 57 gene families potentially acquired through horizontal gene transfer, of which 39 are specific to land plants. Functional characterization of these candidate gene families suggested their implication in various biological processes such as carbohydrate metabolism (e.g. starch biosynthesis or cellulose degradation), reproduction (pollen and seed germination), defense against pathogens (e.g. biosynthesis of polyamines), and abiotic stress tolerance (such as arginase gene involved in adaptation to water shortage, temperature variations and salt stress or genes related to DNA reparation). Interestingly, these horizontally acquired genes had various donors from both different bacterial and fungal groups (Yue et al. 2012).

### Adaptation to UV-induced DNA damages

On land, organisms must face significant higher levels of UV radiation than in water. This stress is known to induce mutagenic and cytotoxic DNA damages. To overcome this risk, plants evolved mechanisms to detect and repair base lesions through, for example, the Base Excision Repair pathway (Fang et al. 2017). In land plants, this pathway is likely initiated by the DNA-3-Methyladenine Glycosylase (MAG). The analysis of *MAG* evolutionary history revealed that plants *MAG* are closer to the bacterial *MAGs* (and form a monophyletic clade) than any other eukaryotic *MAGs*. Interestingly, this gene was also found among Charophyte algae representing the closest relative to land plants (together forming the streptophytes lineage) known to live in brackish water and in aero-terrestrial conditions. Thus, it has been concluded that plant MAGs derived from a single HGT event from bacteria (Fang et al. 2017). Furthermore, an analysis of selective pressure and gene structure revealed a subsequent diversification of *MAG* genes in plants after their horizontal acquisition characterized by a relaxation of the purifying selection shortly after the HGT event and the emergence of introns.

### Key regulatory hubs have been horizontally transferred and predated terrestrialization

Charophytes algae represent the closest lineages of land plants. Among them many are aero-terrestrial and until recently, only few genomes of such algae were available and the *Zygnematophyceae*, sister clade of all land plants, were ignored. The analysis of the first *Zygnematophyceae* genomes revealed that crucial genes involved in multiple fundamental aspects of plant biology results from HGT in the common ancestor of both *Zygnematophyceae* and terrestrial plants. More specifically, 2 gene families were found to have been transferred likely from soil bacteria.

The first corresponds to the GRAS transcription factors which is involved in various process of plant biology such as biotic interactions, abiotic stress tolerance, development, growth, and reproduction (Jaiswal et al. 2022; Waseem et al. 2022). Phylogenetic analysis revealed that GRAS domains are present in bacteria from which 1 subgroup form a monophyletic clade with plants with whom they share the same motif’s structure (Fang et al. 2017). This analysis also suggested a single HGT from bacteria followed by massive expansion in *Zygnematophyceae* and land plants. The second candidate identified through this approach encode the PYL receptor perceiving the abscisic acid and involved in various abiotic stresses tolerance such as desiccation (Jahan et al. 2019). As for the GRAS, plants *PYL* families form a monophyletic clade nested within soil bacterial genes and subsequently diversified into different subfamilies in land plants such as *PYL*/*PYR*/*RCAR* (Cheng et al. 2019).

Overall, these different studies highlight the importance of HGTs in one of the greatest transitions that paved the road to the terrestrialization of other organisms such as animals.

### The vascular system: a land plant functional innovation deriving from HGT

Among the approximate 500,000 extant plant species, around 80% are vascular. The evolution of the vascular system has been a major transition allowing the long-distance transport of metabolites transport and thus the conquest of the heights by plants. This event took place ∼420 MYA in the common ancestor of Lycophytes, ferns, gymnosperms and angiosperms.

The development of vascular tissues is regulated by various genes whose many have suggested to derive from HGT. Among them, the most striking examples are the Transaldolase type A (*TAL-TA*; Yang et al. 2015), the Phenylalanine Ammonia Lyase (*PAL*; Emiliani et al. 2009), and the Vein Patterning 1 genes (*VEP1*; Tarrío et al. 2011). Both *PAL* and *TAL-TA* genes have been acquired in the ancestor of land plants through different routes. In one hand, *PAL* has likely first been transferred from bacteria to the common ancestor of Dikarya fungi and then transferred to plants before the divergence of land plants. In the other hand, *TAL-TA* was likely acquired from actinobacteria and then experienced a relaxation of the selection pressure before the divergence of land plants and the emergence of an intronic-exonic structure (Emiliani et al. 2009; Yang et al. 2015).

Contrary to *TAL-TA* and *PAL* that have been acquired only once, *VEP1* has a more complex history with multiple HGT from bacteria to ancestor of land plants and ancestor of vascular plants (Tarrío et al. 2011). Despite their different evolutionary trajectories, these 3 genes fulfill essential functions in the development of the vascular tissues that have been functionally demonstrated such vein patterning or regulation of vascular bundles development. As they emerged in the common ancestor of land plants and thus are present in non-vascular plants such as the liverwort *Marchantia polymorpha* and the moss *Physcomitrium patens*, it has been hypothesized that they have other functions (e.g. development, response to stresses) in these species and acquired their vascular functions in the common ancestor of vascular plants.

## HGT facilitated the independent evolution of plant immune mechanisms

Genetic studies in angiosperms have identified immune mechanisms deployed by plants in response to pathogens (Jones et al. 2024). The most recent common ancestor of the land plants already possessed intracellular and surface receptors to perceive pathogen-associated molecular patterns and effectors produced by pathogens, as well as the metabolic pathways to produce defense hormones and active specialized chemicals such as phenylpropanoids (Delaux and Schornack 2021). In addition to their conserved immune features, all plant lineages have diversified their immune potential, often via the duplication and neo-functionalization of existing gene families (Delaux and Schornack 2021). In the past years, with the sequencing of a large diversity of plant genomes, the role of HGT in this process has started to emerge.

A common theme is the horizontal transfer of toxins from a diversity of donor species to diverse plant lineages. Cycas belong to the gymnosperms within the seed plant lineage. Within the genus, the *C. panzhihuaensis* genome was sequenced in 2022 and a gene encoding for a putative insecticidal toxin (*FitD*) was identified (Liu et al. 2022). Genomic and phylogenetic analyses revealed the bacterial origin of *FitD* that was horizontally transferred from a bacterium, possibly from the *Pseudomonas*genus (Liu et al. 2022). Using injection assays of the synthetized FitD revealed its insecticidal effect against 2 pathogens, the moth *Plutella xylostella* and the cotton bollworm *Helicoverpa armigera* (Liu et al. 2022). The number of *FitD* copies varies from one Cycas species to another, suggesting that the gene experienced duplications following the original transfer. Whether all copies encode toxins with the same biological function, and pathogen targets, remains to be determined.

Another example of HGT-mediated gain of toxin occurred in ferns. The first available fern genomes, the aquatic ferns *Azolla filiculoides and Salvinia cucullata*, revealed 1 gene of bacterial origin *Tma12* (Li et al. 2018a). Sequence-based annotation and study of its structure suggest that *Tma12* encodes for a putative lytic polysaccharide monooxygenase (Yadav et al. 2019). *Tma12* was originally identified from a bioassay-based purification approach searching for insecticidal peptides and proteins (Shukla et al. 2016). Besides its in vitro demonstrated insecticidal properties, transferring by transgenesis *Tma12* from the fern *Tectaria macrodonta* to cotton provided resistance against Whitefly (*Bemisia tabaci*) (Shukla et al. 2016). Interestingly, from a history of science perspective, the technology-assisted HGT from ferns to cotton predated the discovery of the origin of *Tma12* in fern from HGT. In a way, this process mimicked what happened millions of years earlier between bacteria and plants.

While in the 2 examples presented above, *FitD* and *Tma12*, the HGT directly conferred the resistance function as the protein itself is insecticidal, improved resistance also evolved indirectly. Liverworts are well known for the very diverse array of specialized metabolites they produce, such as terpenoids (Ludwiczuk and Asakawa 2019). Among the 39 terpene synthase-encoding genes in the latest genome assembly of the model liverwort *Marchantia polymorpha* Tak-1, 32 are annotated as microbial terpene synthase-like (MTPSL). Preliminary survey of plant transcriptomes had revealed that these MTPSL likely originated by HGT from bacteria and fungi (Jia et al. 2016), a finding confirmed for the fungal-derived ones using an expanded dataset including a large diversity of plant genomes (Mahboubi et al. 2024). From this study, it can be inferred that the transfer occurred from an ancestor of the extant Dykaria lineage to an ancestor of extant bryophytes (Mahboubi et al. 2024). Biochemical assays on several of these HGT-derived MTPSL from bryophytes revealed their ability to produce diverse terpenes (Jia et al. 2016; Kumar et al. 2016; Takizawa et al. 2021). RNAi targeting MTPSL1/2/4/9/10/5 led to a quantitative decrease in the blend of terpene synthesized by *M. polymorpha*, and a very minor effect on the feeding efficiency of neonates from the generalist herbivore *Spodoptera litura* (Takizawa et al. 2021). Due to the diversification of the MTPSL following the original HGT, it can be anticipated that the validation of the functional role of these MTPSL by reverse genetics will be a challenge. However, a recent link between MTPSL and resistance has been drawn using forward genetics. A diversity of populations from *M. polymorpha* has been collected and sequenced, leading to an intra-specific diversity resource for this liverwort (Beaulieu et al. 2025; Wu et al. 2025). Using this resource, El Mahboubi et al. conducted a Genome-Wide Association Study on the resistance/susceptibility of *M. polymorpha* to a fungal pathogen, *Colletotrichum nymphaeae* (Mahboubi et al. 2024). While quantitative resistance to *C. nymphaeae* seems to be a polygenic trait in *M. polymorpha*, 1 locus stood out, encompassing a cluster of 4 MTPSL in the M*. polymorpha* Tak-1 reference genome (Mahboubi et al. 2024). The number of copies, or even the presence of this cluster, seems to differ between *M. polymorpha* accessions, with susceptible accessions often missing the entire genomic region (Mahboubi et al. 2024). In addition, surveying the *M. polymorpha* pangenome revealed another gene associated to climate adaptation which originated from HGT, a Fungal Fruit Body Lectin (Beaulieu et al. 2025), likely transferred from a fungus to the most recent common ancestor of the land plants and subsequently lost in angiosperms (Peumans et al. 2007; Beaulieu et al. 2025). This finding highlights that HGT may provide material for continuous adaptation to occur, one of the components of the arms race between host and pathogens (Brown and Tellier 2011).

### HGTs are active players in the arms race between plant hosts and pathogens

The gain of toxins via HGT, either toxic proteins or specialized metabolites originating from new enzymatic abilities, and the role of these toxins in resistance sets the scene for another layer of arms race with the insect or microbial pathogens they target. Conceptually, it can be anticipated that resistance to these toxins may evolve by HGT as well, similar to the gain of antibiotic resistance in bacteria via the transfer of plasmids (MacLean and San Millan 2019). Two examples illustrate this concept.

Insects are generally sensitive to phenolic glycoside produced by many plant species (Xia et al. 2021). However, Whiteflies are not. Xia et al. identified 1 gene sufficient to confer full resistance to these toxic compounds, the malonyltransferase gene *BtMaT1*, a gene that originated from an HGT from plants. Silencing of this gene using host-induced gene silencing was sufficient to confer full resistance to whiteflies in tomato. A scenario emerges in which the basal insect defense provided by phenolic glycoside was successfully overcame by whiteflies through the HGT of *MaT1*, while ferns went 1 step further in this arms race by gaining the toxin *Tma12* by HGT from bacteria. Reciprocally, and filling the gap in the HGT-mediated arms race loop, HGT has also provided resistance to toxins in plants. The GST *Fhb7* was transferred horizontally from fungi of an *Epichloë* species to grasses, providing resistance to Fusarium head blight in wheat (Wang et al. 2020a).

## HGT facilitated the establishment of symbiotic mutualism

The vast diversity of organisms within the green lineage is accompanied by a wide array of mutualistic interactions with microbes, including bacteria and fungi. HGTs are believed to have played a crucial role in the evolution of mutualism, particularly on the microbial side.

### HGT in microbial symbionts contributed to the evolution of mycorrhizal and rhizobial symbioses

For example, in the nitrogen-fixing root nodule symbiosis (RNS) between legumes and rhizobia, HGT significantly shaped the symbiotic relationship. The transfer of bacterial symbiotic plasmids (Clerissi et al. 2018) and symbiotic islands (Ling et al. 2016; Bamba et al. 2020)—large genomic regions containing essential symbiosis-related genes—allowed diverse rhizobia lineages to gain the ability to engage with RNS host, to switch to alternative hosts and, in a lab-induced HGT, to transition from a pathogenic lifestyle to an endosymbiotic one (Remigi et al. 2014).

In plant-fungi mutualisms, such as the arbuscular mycorrhizal (AM) and ectomycorrhizal (ECM) symbioses, HGT has also influenced fungal partners. For instance, genes involved in gene regulation, mitosis, and signal transduction were horizontally transferred from plants and bacteria to the AM fungus *Rhizophagus irregularis* (Li et al. 2018b). Another example is the transfer of ACC deaminase genes to ECM fungi of the *Amanita* genus, where these genes are highly up-regulated during symbiosis and are believed to modulate the plant's immune response (Wang et al. 2020b). Collectively, these HGT events may have contributed to the evolution and adaptation of mycorrhizal symbioses.

### HGTs contributed to the evolution of lichens in algal symbionts

While the above examples illustrate the impact of HGT on microbial symbionts, there is one mutualism in which the plant partners themselves acquired crucial genes through HGT—the lichen symbiosis. Lichens are symbiotic structures composed of a fungal partner and a photosynthetic organism, which can be either a cyanobacterium or, more commonly, a green alga. These lichen algal symbionts (LAS) belong to the green lineage (Viridiplantae), specifically within the Chlorophytes (Fig. 4). Within the chlorophyte phylum, LAS are found in 2 classes: *Ulvophyceae* and *Trebouxiophyceae* (Sanders and Masumoto 2021; Spribille et al. 2022). Lichenization has evolved multiple times independently in Chlorophytes, making it a clear example of convergent evolution (Lutzoni et al. 2018; Puginier et al. 2024). The evolution of lichen symbiosis in LAS was, in part, facilitated by horizontal gene transfers HGTs. Genomic and transcriptomic analyses of LAS have revealed that several genes enhancing algal resistance to desiccation and oxidative stress were horizontally acquired (Carniel et al. 2016; Puginier et al. 2024), thereby improving adaptation to the aeroterrestrial lifestyle. For instance, a glutathione S-transferase (*GST*) was horizontally transferred from bacteria to symbiotic *Trebouxiophyceae* (Fig. 4). GST enzymes have previously been identified in lichen symbionts as key players in oxidative stress resistance, leading to improved desiccation tolerance (Kranner et al. 2005). Similarly, transcriptome analysis of *Trebouxia gelatinosa*, a LAS from the *Trebouxiophyceae*, identified an expanded gene family known as desiccation-related proteins (*DRPs*), which were likely acquired through HGT from bacteria inhabiting lichen thalli (Fig. 4). The acquisition and expansion of DRPs are thought to further enhance desiccation tolerance (Carniel et al. 2016). HGT events between fungi and LAS have also been documented. A study found that 3 genes—Tellurite-resistance dicarboxylase (*TDT*), Nitrilase/cyanide hydratase (*CH*), and Oxidoreductase/retinol dehydrogenase (*OD*)—were transferred from Ascomycete fungi to the LAS *Trebouxia decolorans* (Beck et al. 2015) (Fig. 4). However, further research is needed to determine the specific functions of these genes and whether they played a role in the emergence of mutualism.

Another notable HGT event involved a carbohydrate active enzyme (CAZY) from the glycoside hydrolase 8 (*GH8*) family, which was transferred from bacteria to *Trebouxiophyceae* (Fig. 4) in parallel with the emergence of lichen symbiosis (Puginier et al. 2024). The enzymatic activity of this algal GH8 enzyme was further investigated, revealing its ability to degrade lichenan, a polymer found in the extracellular matrix of lichen fungal symbionts (Honegger and Haisch 2001). Interestingly, previous studies have shown that this fungal extracellular matrix is less prominent in the presence of LAS (Honegger and Haisch 2001), which aligns with the identification of an algal enzyme capable of breaking down one of its components. From this point, we can hypothesize that the GH8 enzyme was acquired and maintained in LAS because it provided an advantage for the mutualism—either by facilitating the establishment of a symbiotic interface that enhances exchanges between lichen symbionts or by aiding in the accommodation of algal symbionts within lichen thalli (Puginier et al. 2024).

## Conclusions

Generating long-read-based genome assemblies across the diversity of plants has allowed identifying hundreds of HGTs across the green lineage, with donor species ranging from bacteria to virus or fungi. The combination of phylogeny- and sequence-based approaches validated part of these events, and in some instances allowed to define an approximate time for the transfer. Genome-wise association studies, reverse genetics and artificial gene transfer confirmed the adaptive role played by Horizontally transmitted genes in plants. Although our understanding of HGT in plants improved over the last decade, a number of open questions and challenges remain.

Our view of HGT in plants is limited by the number of available datasets. There is no doubt that large-scale sequencing projects currently generating high-quality genome assemblies covering the entire green lineage will allow defining the global map of past HGT within the plant kingdom. Combined with lifestyle, ecological niches, past and extant climatic data, this global view will enable associating the prevalence of HGT with selection pressure, as recently done for the adaptation to special lifestyle and habitats which resulted in a convergent reduction in immune receptors (Li et al. 2025).

The mechanisms underlying HGT in plants, and in eukaryotes more broadly, remain poorly understood. Referring to these as “mechanisms” can be misleading, as HGT is not a single well-defined process but rather a series of molecular events. Understanding how nuclear genes can be successfully transferred between species remains a major challenge in plant biology. Researchers face a kind of “footprints-not-footsteps” challenge. In other words, by the time an HGT event is detected, the process itself has already occurred. Like arriving on a battlefield after the fighting has ended, we can observe only the outcome—the traces of foreign genetic material integrated into a new species—but not sequence of events that led to its incorporation into the recipient species' genome, nor the mechanism by which the genetic material exited the donor species. The molecular pathways, the barriers overcome, and the forces that facilitated the transfer will remain unknown. Consequently, we can only formulate hypotheses about the sequence of events based on prior knowledge, leaving a significant gap in our understanding of the “mechanisms” of HGT.

Above all challenges, catching an ongoing HGT, in an environmental niche where it just happened and did not yet spread within a population of plants, remains a main endeavor. Such event would provide access to the donor species, facilitate the identification of the transfer mechanism, and at the same time would allow describing in real time its adaptive potential. Plant—plant HGT are particularly attractive in that context as both the donor and the recipient species are accessible.

The impact of HGT on plant functional evolution has been rarely elucidated, mostly relying on indirect evidence, such as phylogenetic correlations (Puginier et al. 2024), in vitro assays (Liu et al. 2022), or GWAS (Mahboubi et al. 2024). The most convincing examples came by mimicking these HGT events, such as the transfer of *Tma12* to provide resistance to insects in cotton (Shukla et al. 2016) or, in bacteria, by transferring entire plasmid to kick start the evolution of symbiotic mutualism (Marchetti et al. 2010). Combining synthetic biology and experimental evolution may thus offer a unique opportunity in the future to replay the evolutionary tape, starting with artificial HGT to project plants in new fitness landscapes.

Finally, the very fact that ancient HGT can be detected implies that they have adaptive potential and that this potential can be rapidly deployed by the recipient genome. In other words, ancient HGT likely provided benefit to the recipient species as single-gene unit following their transfer. Single-gene traits represent an opportunity for crop improvement. The transfer of resistance to whiteflies provided by *Tma12* or the introduction of *CSP*—which increases drought tolerance and ultimately yield in wheat—illustrates this concept. The number of known genes originating from HGT has massively expanded over the last years due to the availability of new, long-read based, genome assemblies. One can predict that the avalanche of new genomes combined with efficient methods to detect HGT will result in the identification of more HGT and thus lists of candidate genes for crop improvement.

## Supplementary Material

koaf195_Supplementary_Data

## Data Availability

No new data were generated or analysed in support of this research.

## Dive Curated Terms

The following phenotypic, genotypic, and functional terms are of significance to the work described in this paper:

LAS Gramene: AT1G55580
LAS Araport: AT1G55580

TDT Gramene: AT5G47560
TDT Araport: AT5G47560

VEP1 Gramene: AT4G24220
VEP1 Araport: AT4G24220

## References

[koaf195-B1] Acuña R, Padilla BE, Flórez-Ramos CP, Rubio JD, Herrera JC, Benavides P, Lee S-J, Yeats TH, Egan AN, Doyle JJ, et al Adaptive horizontal transfer of a bacterial gene to an invasive insect pest of coffee. Proc Natl Acad Sci U S A. 2012:109(11):4197–4202. 10.1073/pnas.1121190109 22371593 PMC3306691

[koaf195-B2] Adato O, Ninyo N, Gophna U, Snir S. Detecting horizontal gene transfer between closely related taxa. PLoS Comput Biol. 2015:11(10):e1004408. 10.1371/journal.pcbi.1004408 26439115 PMC4595140

[koaf195-B3] Ali A, Bang SW, Chung S-M, Staub JE. Plant transformation via pollen tube-mediated gene transfer. Plant Mol Biol Rep. 2015:33(3):742–747. 10.1007/s11105-014-0839-5

[koaf195-B4] Anderson JV, Chao WS, Horvath DP. A current review on the regulation of dormancy in vegetative buds. Weed Sci. 2001:49(5):581–589. 10.1614/0043-1745(2001)049[0581:RCROTR]2.0.CO;2

[koaf195-B5] Arnold BJ, Huang I-T, Hanage WP. Horizontal gene transfer and adaptive evolution in bacteria. Nat Rev Microbiol. 2022:20(4):206–218. 10.1038/s41579-021-00650-4 34773098

[koaf195-B6] Aubin E, El Baidouri M, Panaud O. Horizontal gene transfers in plants. Life. 2021:11(8):857. 10.3390/life11080857 34440601 PMC8401529

[koaf195-B7] Aubin E, Llauro C, Garrigue J, Mirouze M, Panaud O, Baidouri ME. Genome-wide analysis of horizontal transfer in non-model wild species from a natural ecosystem reveals new insights into genetic exchange in plants. PLoS Genet. 2023:19(10):e1010964. 10.1371/journal.pgen.1010964 37856455 PMC10586619

[koaf195-B8] Baidouri ME, Carpentier M-C, Cooke R, Gao D, Lasserre E, Llauro C, Mirouze M, Picault N, Jackson SA, Panaud O. Widespread and frequent horizontal transfers of transposable elements in plants. Genome Res. 2014:24(5):831–838. 10.1101/gr.164400.113 24518071 PMC4009612

[koaf195-B9] Balios VA, Fischer K, Bawin T, Krause K. One organ to infect them all: the *Cuscuta* haustorium. Ann Bot. 2025:135(5):823–840. 10.1093/aob/mcae208 39673400 PMC12064427

[koaf195-B10] Bamba M, Aoki S, Kajita T, Setoguchi H, Watano Y, Sato S, Tsuchimatsu T. Massive rhizobial genomic variation associated with partner quality in *Lotus–Mesorhizobium* symbiosis. FEMS Microbiol Ecol. 2020:96(12):fiaa202. 10.1093/femsec/fiaa202 33016310

[koaf195-B11] Bansal MS, Kellis M, Kordi M, Kundu S. RANGER-DTL 2.0: rigorous reconstruction of gene-family evolution by duplication, transfer and loss. Bioinformatics. 2018:34(18):3214–3216. 10.1093/bioinformatics/bty314 29688310 PMC6137995

[koaf195-B12] Beaulieu C, Libourel C, Mbadinga Zamar DL, El Mahboubi K, Hoey DJ, Greiff GRL, Keller J, Girou C, San Clemente H, Diop I, et al The Marchantia polymorpha pangenome reveals ancient mechanisms of plant adaptation to the environment. Nat Genet. 2025:57(3):729–740. 10.1038/s41588-024-02071-4 39962240 PMC11906373

[koaf195-B13] Beck A, Divakar PK, Zhang N, Molina MC, Struwe L. Evidence of ancient horizontal gene transfer between fungi and the terrestrial alga Trebouxia. Org Divers Evol. 2015:15(2):235–248. 10.1007/s13127-014-0199-x

[koaf195-B14] Belser C, Istace B, Denis E, Dubarry M, Baurens F-C, Falentin C, Genete M, Berrabah W, Chèvre A-M, Delourme R, et al Chromosome-scale assemblies of plant genomes using nanopore long reads and optical maps. Nat Plants. 2018:4(11):879–887. 10.1038/s41477-018-0289-4 30390080

[koaf195-B15] Bock R . The give-and-take of DNA: horizontal gene transfer in plants. Trends Plant Sci. 2010:15(1):11–22. 10.1016/j.tplants.2009.10.001 19910236

[koaf195-B16] Brown JKM, Tellier A. Plant-parasite coevolution: bridging the gap between genetics and ecology. Annu Rev Phytopathol. 2011:49(1):345–367. 10.1146/annurev-phyto-072910-095301 21513455

[koaf195-B17] Campbell WH, Gowri G. Codon usage in higher plants, green Algae, and Cyanobacteria. Plant Physiol. 1990:92(1):1–11. 10.1104/pp.92.1.1 16667228 PMC1062239

[koaf195-B18] Carniel FC, Gerdol M, Montagner A, Banchi E, De Moro G, Manfrin C, Muggia L, Pallavicini A, Tretiach M. New features of desiccation tolerance in the lichen photobiont Trebouxia gelatinosa are revealed by a transcriptomic approach. Plant Mol Biol. 2016:91(3):319–339. 10.1007/s11103-016-0468-5 26992400

[koaf195-B19] Chakraborty S, Yengkhom S, Uddin A. Analysis of codon usage bias of chloroplast genes in Oryza species. Planta. 2020:252(4):67. 10.1007/s00425-020-03470-7 32989601

[koaf195-B20] Cheng S, Xian W, Fu Y, Marin B, Keller J, Wu T, Sun W, Li X, Xu Y, Zhang Y, et al Genomes of subaerial Zygnematophyceae provide insights into land plant evolution. Cell. 2019:179(5):1057–1067.e14. 10.1016/j.cell.2019.10.019 31730849

[koaf195-B21] Christin P-A, Edwards EJ, Besnard G, Boxall SF, Gregory R, Kellogg EA, Hartwell J, Osborne CP. Adaptive evolution of C4 photosynthesis through recurrent lateral gene transfer. Curr Biol. 2012:22(5):445–449. 10.1016/j.cub.2012.01.054 22342748

[koaf195-B22] Clerissi C, Touchon M, Capela D, Tang M, Cruveiller S, Genthon C, Lopez-Roques C, Parker MA, Moulin L, Masson-Boivin C, et al Parallels between experimental and natural evolution of legume symbionts. Nat Commun. 2018:9(1):2264. 10.1038/s41467-018-04778-5 29891837 PMC5995829

[koaf195-B23] David-Schwartz R, Runo S, Townsley B, Machuka J, Sinha N. Long-distance transport of mRNA via parenchyma cells and phloem across the host–parasite junction in *Cuscuta*. New Phytol. 2008:179(4):1133–1141. 10.1111/j.1469-8137.2008.02540.x 18631294

[koaf195-B24] Davis CC, Wurdack KJ. Host-to-parasite gene transfer in flowering plants: phylogenetic evidence from Malpighiales. Science. 2004:305(5684):676–678. 10.1126/science.1100671 15256617

[koaf195-B25] Davis CC, Xi Z. Horizontal gene transfer in parasitic plants. Curr Opin Plant Biol. 2015:26:14–19. 10.1016/j.pbi.2015.05.008 26051213

[koaf195-B26] Delaux P-M, Schornack S. Plant evolution driven by interactions with symbiotic and pathogenic microbes. Science. 2021:371(6531):eaba6605. 10.1126/science.aba6605 33602828

[koaf195-B27] de Tomás C, Vicient CM. Genome-wide identification of reverse transcriptase domains of recently inserted endogenous plant pararetrovirus (Caulimoviridae). Front Plant Sci. 2022:13:1011565. 10.3389/fpls.2022.1011565 36589050 PMC9794742

[koaf195-B28] Dickinson TA . Epiphylly in angiosperms. Bot Rev. 1978:44(2):181–232. 10.1007/BF02919079

[koaf195-B29] Dunning LT, Olofsson JK, Parisod C, Choudhury RR, Moreno-Villena JJ, Yang Y, Dionora J, Quick WP, Park M, Bennetzen JL, et al Lateral transfers of large DNA fragments spread functional genes among grasses. Proc Natl Acad Sci U S A. 2019:116(10):4416–4425. 10.1073/pnas.1810031116 30787193 PMC6410850

[koaf195-B30] Emiliani G, Fondi M, Fani R, Gribaldo S. A horizontal gene transfer at the origin of phenylpropanoid metabolism: a key adaptation of plants to land. Biol Direct. 2009:4(1):7. 10.1186/1745-6150-4-7 19220881 PMC2657906

[koaf195-B31] Fang H, Huangfu L, Chen R, Li P, Xu S, Zhang E, Cao W, Liu L, Yao Y, Liang G, et al Ancestor of land plants acquired the DNA-3-methyladenine glycosylase (MAG) gene from bacteria through horizontal gene transfer. Sci Rep. 2017:7(1):9324. 10.1038/s41598-017-05066-w 28839126 PMC5570899

[koaf195-B32] Friedman R, Ely B. Codon usage methods for horizontal gene transfer detection generate an abundance of false positive and false negative results. Curr Microbiol. 2012:65(5):639–642. 10.1007/s00284-012-0205-5 23010940

[koaf195-B33] Geering ADW, Maumus F, Copetti D, Choisne N, Zwickl DJ, Zytnicki M, McTaggart AR, Scalabrin S, Vezzulli S, Wing RA, et al Endogenous florendoviruses are major components of plant genomes and hallmarks of virus evolution. Nat Commun. 2014:5(1):5269. 10.1038/ncomms6269 25381880 PMC4241990

[koaf195-B34] Gilbert C, Chateigner A, Ernenwein L, Barbe V, Bézier A, Herniou EA, Cordaux R. Population genomics supports baculoviruses as vectors of horizontal transfer of insect transposons. Nat Commun. 2014:5(1):3348. 10.1038/ncomms4348 24556639 PMC3948050

[koaf195-B35] Gilbert C, Maumus F. Multiple horizontal acquisitions of plant genes in the whitefly *Bemisia tabaci*. Genome Biol Evol. 2022:14(10):evac141. 10.1093/gbe/evac141 36155788 PMC9599486

[koaf195-B36] Gilbert C, Schaack S, Pace JK II, Brindley PJ, Feschotte C. A role for host–parasite interactions in the horizontal transfer of transposons across phyla. Nature. 2010:464(7293):1347–1350. 10.1038/nature08939 20428170 PMC3004126

[koaf195-B37] Goldschmidt Eliezer E. Plant grafting: new mechanisms, evolutionary implications. Front Plant Sci. 2014:5. 10.3389/fpls.2014.00727 PMC4269114 25566298

[koaf195-B38] Herbst J, Li Q-Q, De Veylder L. Mechanistic insights into DNA damage recognition and checkpoint control in plants. Nat Plants. 2024:10(4):539–550. 10.1038/s41477-024-01652-9 38503962

[koaf195-B39] Hibdige SGS, Raimondeau P, Christin P-A, Dunning LT. Widespread lateral gene transfer among grasses. New Phytol. 2021:230(6):2474–2486. 10.1111/nph.17328 33887801

[koaf195-B40] Hill T, Nordström KJ, Thollesson M, Säfström TM, Vernersson AK, Fredriksson R, Schiöth HB. SPRIT: identifying horizontal gene transfer in rooted phylogenetic trees. BMC Evol Biol. 2010:10(1):42. 10.1186/1471-2148-10-42 20152048 PMC2829038

[koaf195-B41] Honegger R, Haisch A. Immunocytochemical location of the (1→3) (1→4)-β-glucan lichenin in the lichen-forming ascomycete *Cetraria islandica* (Icelandic moss)^1^. New Phytol. 2001:150(3):739–746. 10.1046/j.1469-8137.2001.00122.x

[koaf195-B42] Jahan A, Komatsu K, Wakida-Sekiya M, Hiraide M, Tanaka K, Ohtake R, Umezawa T, Toriyama T, Shinozawa A, Yotsui I, et al Archetypal roles of an abscisic acid receptor in drought and sugar responses in liverworts. Plant Physiol. 2019:179(1):317–328. 10.1104/pp.18.00761 30442644 PMC6324230

[koaf195-B43] Jaiswal V, Kakkar M, Kumari P, Zinta G, Gahlaut V, Kumar S. Multifaceted roles of GRAS transcription factors in growth and stress responses in plants. iScience. 2022:25(9):105026. 10.1016/j.isci.2022.105026 36117995 PMC9474926

[koaf195-B44] Jia Q, Li G, Köllner TG, Fu J, Chen X, Xiong W, Crandall-Stotler BJ, Bowman JL, Weston DJ, Zhang Y, et al Microbial-type terpene synthase genes occur widely in nonseed land plants, but not in seed plants. Proc Natl Acad Sci U S A. 2016:113(43):12328–12333. 10.1073/pnas.1607973113 27791023 PMC5087002

[koaf195-B45] Jiao W-B, Accinelli GG, Hartwig B, Kiefer C, Baker D, Severing E, Willing E-M, Piednoel M, Woetzel S, Madrid-Herrero E, et al Improving and correcting the contiguity of long-read genome assemblies of three plant species using optical mapping and chromosome conformation capture data. Genome Res. 2017:27(5):778–786. 10.1101/gr.213652.116 28159771 PMC5411772

[koaf195-B46] Jones JDG, Staskawicz BJ, Dangl JL. The plant immune system: from discovery to deployment. Cell. 2024:187(9):2095–2116. 10.1016/j.cell.2024.03.045 38670067

[koaf195-B47] Kado T, Innan H. Horizontal gene transfer in five parasite plant species in Orobanchaceae. Genome Biol Evol. 2018:10(12):3196–3210. 10.1093/gbe/evy219 30407540 PMC6294234

[koaf195-B48] Kim G, LeBlanc ML, Wafula EK, dePamphilis CW, Westwood JH. Genomic-scale exchange of mRNA between a parasitic plant and its hosts. Science. 2014:345(6198):808–811. 10.1126/science.1253122 25124438

[koaf195-B49] Kitagawa M, Tran TM, Jackson D. Traveling with purpose: cell-to-cell transport of plant mRNAs. Trends Cell Biol. 2024:34(1):48–57. 10.1016/j.tcb.2023.05.010 37380581

[koaf195-B50] Koo D-H, Molin WT, Saski CA, Jiang J, Putta K, Jugulam M, Friebe B, Gill BS. Extrachromosomal circular DNA-based amplification and transmission of herbicide resistance in crop weed *Amaranthus palmeri*. Proc Natl Acad Sci U S A. 2018:115(13):3332–3337. 10.1073/pnas.1719354115 29531028 PMC5879691

[koaf195-B51] Kranner I, Cram WJ, Zorn M, Wornik S, Yoshimura I, Stabentheiner E, Pfeifhofer HW. Antioxidants and photoprotection in a lichen as compared with its isolated symbiotic partners. Proc Natl Acad Sci U S A. 2005:102(8):3141–3146. 10.1073/pnas.0407716102 15710882 PMC549463

[koaf195-B52] Kumar S, Kempinski C, Zhuang X, Norris A, Mafu S, Zi J, Bell SA, Nybo SE, Kinison SE, Jiang Z, et al Molecular diversity of terpene synthases in the liverwort Marchantia polymorpha. Plant Cell. 2016:28(10):2632–2650. 10.1105/tpc.16.00062 27650333 PMC5134972

[koaf195-B53] Kyndt T, Quispe D, Zhai H, Jarret R, Ghislain M, Liu Q, Gheysen G, Kreuze JF. The genome of cultivated sweet potato contains *Agrobacterium* T-DNAs with expressed genes: an example of a naturally transgenic food crop. Proc Natl Acad Sci U S A. 2015:112(18):5844–5849. 10.1073/pnas.1419685112 25902487 PMC4426443

[koaf195-B54] Lanciano S, Carpentier M-C, Llauro C, Jobet E, Robakowska-Hyzorek D, Lasserre E, Ghesquière A, Panaud O, Mirouze M. Sequencing the extrachromosomal circular mobilome reveals retrotransposon activity in plants. PLoS Genet. 2017:13(2):e1006630. 10.1371/journal.pgen.1006630 28212378 PMC5338827

[koaf195-B55] Lapadula WJ, Mascotti ML, Juri Ayub M. Whitefly genomes contain ribotoxin coding genes acquired from plants. Sci Rep. 2020:10(1):15503. 10.1038/s41598-020-72267-1 32968092 PMC7511414

[koaf195-B56] Laroche J, Li P, Maggia L, Bousquet J. Molecular evolution of angiosperm mitochondrial introns and exons. Proc Natl Acad Sci U S A. 1997:94(11):5722–5727. 10.1073/pnas.94.11.5722 9159140 PMC20846

[koaf195-B57] Lausser A, Kliwer I, Srilunchang K, Dresselhaus T. Sporophytic control of pollen tube growth and guidance in maize. J Exp Bot. 2010:61(3):673–682. 10.1093/jxb/erp330 19926683 PMC2814102

[koaf195-B58] Li F-W, Brouwer P, Carretero-Paulet L, Cheng S, de Vries J, Delaux P-M, Eily A, Koppers N, Kuo L-Y, Li Z, et al Fern genomes elucidate land plant evolution and cyanobacterial symbioses. Nat Plants. 2018a:4(7):460–472. 10.1038/s41477-018-0188-8 29967517 PMC6786969

[koaf195-B59] Li F-W, Villarreal JC, Kelly S, Rothfels CJ, Melkonian M, Frangedakis E, Ruhsam M, Sigel EM, Der JP, Pittermann J, et al Horizontal transfer of an adaptive chimeric photoreceptor from bryophytes to ferns. Proc Natl Acad Sci U S A. 2014:111(18):6672–6677. 10.1073/pnas.1319929111 24733898 PMC4020063

[koaf195-B60] Li L, Peng S, Wang Z, Zhang T, Li H, Xiao Y, Li J, Liu Y, Yin H. Genome mining reveals abiotic stress resistance genes in plant genomes acquired from microbes via HGT. Front Plant Sci. 2022:13:1025122. 10.3389/fpls.2022.1025122 36407614 PMC9667741

[koaf195-B61] Li M, Zhao J, Tang N, Sun H, Huang J. Horizontal gene transfer from Bacteria and plants to the arbuscular mycorrhizal fungus Rhizophagus irregularis. Front Plant Sci. 2018b:9:701. 10.3389/fpls.2018.00701 29887874 PMC5982333

[koaf195-B62] Li S-X, Liu Y, Zhang Y-M, Chen J-Q, Shao Z-Q. Convergent reduction of immune receptor repertoires during plant adaptation to diverse special lifestyles and habitats. Nat Plants. 2025:11(2):248–262. 10.1038/s41477-024-01901-x 39821112

[koaf195-B63] Ling J, Wang H, Wu P, Li T, Tang Y, Naseer N, Zheng H, Masson-Boivin C, Zhong Z, Zhu J. Plant nodulation inducers enhance horizontal gene transfer of *Azorhizobium caulinodans* symbiosis island. Proc Natl Acad Sci U S A. 2016:113(48):13875–13880. 10.1073/pnas.1615121113 27849579 PMC5137767

[koaf195-B64] Liu Y, Wang S, Li L, Yang T, Dong S, Wei T, Wu S, Liu Y, Gong Y, Feng X, et al The Cycas genome and the early evolution of seed plants. Nat Plants. 2022:8(4):389–401. 10.1038/s41477-022-01129-7 35437001 PMC9023351

[koaf195-B65] Long M, de Souza SJ, Gilbert W. Evolution of the intron-exon structure of eukaryotic genes. Curr Opin Genet Dev. 1995:5(6):774–778. 10.1016/0959-437X(95)80010-3 8745076

[koaf195-B66] Ludwiczuk A, Asakawa Y. Bryophytes as a source of bioactive volatile terpenoids—a review. Food Chem Toxicol. 2019:132:110649. 10.1016/j.fct.2019.110649 31254593

[koaf195-B67] Luo K-R, Huang N-C, Yu T-S. Selective targeting of Mobile mRNAs to plasmodesmata for cell-to-cell movement. Plant Physiol. 2018:177(2):604–614. 10.1104/pp.18.00107 29581179 PMC6001314

[koaf195-B68] Lutzoni F, Nowak MD, Alfaro ME, Reeb V, Miadlikowska J, Krug M, Arnold AE, Lewis LA, Swofford DL, Hibbett D, et al Contemporaneous radiations of fungi and plants linked to symbiosis. Nat Commun. 2018:9(1):5451. 10.1038/s41467-018-07849-9 30575731 PMC6303338

[koaf195-B69] Ma J, Wang S, Zhu X, Sun G, Chang G, Li L, Hu X, Zhang S, Zhou Y, Song C-P, et al Major episodes of horizontal gene transfer drove the evolution of land plants. Mol Plant. 2022:15(5):857–871. 10.1016/j.molp.2022.02.001 35235827

[koaf195-B70] Ma Z, Yin X, Chang D, Hu X, Boye JI. Long- and short-range structural characteristics of pea starch modified by autoclaving, α-amylolysis, and pullulanase debranching. Int J Biol Macromol. 2018:120(Pt A):650–656. 10.1016/j.ijbiomac.2018.08.132 30165145

[koaf195-B71] MacLean RC, San Millan A. The evolution of antibiotic resistance. Science. 2019:365(6458):1082–1083. 10.1126/science.aax3879 31515374

[koaf195-B72] Mahboubi KE, Beaulieu C, Castel B, Libourel C, Jariais N, Amblard E, van Beveren F, Keller J, Martinez Y, Nelson J, et al Horizontal gene transfers and terpene metabolism drive plant-fungal interaction in *Marchantia polymorpha*. bioRxiv 2024. 10.1101/2024.12.20.629586, preprint: not peer reviewed. PMC12890914 41637459

[koaf195-B73] Mahelka V, Krak K, Kopecký D, Fehrer J, Šafář J, Bartoš J, Hobza R, Blavet N, Blattner FR. Multiple horizontal transfers of nuclear ribosomal genes between phylogenetically distinct grass lineages. Proc Natl Acad Sci U S A. 2017:114(7):1726–1731. 10.1073/pnas.1613375114 28137844 PMC5320982

[koaf195-B74] Marchant DB, Chen G, Cai S, Chen F, Schafran P, Jenkins J, Shu S, Plott C, Webber J, Lovell JT, et al Dynamic genome evolution in a model fern. Nat Plants. 2022:8(9):1038–1051. 10.1038/s41477-022-01226-7 36050461 PMC9477723

[koaf195-B75] Marchetti M, Capela D, Glew M, Cruveiller S, Chane-Woon-Ming B, Gris C, Timmers T, Poinsot V, Gilbert LB, Heeb P, et al Experimental evolution of a plant pathogen into a legume symbiont. PLoS Biol. 2010:8(1):e1000280. 10.1371/journal.pbio.1000280 20084095 PMC2796954

[koaf195-B76] Menezes-e-Vasconcelos K, Melo-de-Pinna GFA. Adventitious buds on roots of Siphanthera arenaria (DC.) Cogn. (Melastomataceae), an annual plant from the cerrado biome. Folia Geobot. 2024:58(3-4):311–315. 10.1007/s12224-023-09433-6

[koaf195-B77] Michael TP, VanBuren R. Building near-complete plant genomes. Curr Opin Plant Biol. 2020:54:26–33. 10.1016/j.pbi.2019.12.009 31981929

[koaf195-B78] Morel B, Schade P, Lutteropp S, Williams TA, Szöllősi GJ, Stamatakis A. SpeciesRax: a tool for Maximum likelihood Species tree inference from gene family trees under duplication, transfer, and loss. Mol Biol Evol. 2022:39(2):msab365. 10.1093/molbev/msab365 35021210 PMC8826479

[koaf195-B79] Mower JP, Stefanović S, Young GJ, Palmer JD. Plant genetics: gene transfer from parasitic to host plants. Nature. 2004:432(7014):165–166. 10.1038/432165b 15538356

[koaf195-B80] Murray EE, Lotzer J, Eberle M. Codon usage in plant genes. Nucleic Acids Res. 1989:17(2):477–498. 10.1093/nar/17.2.477 2644621 PMC331598

[koaf195-B81] Nikolaidis N, Doran N, Cosgrove DJ. Plant expansins in bacteria and fungi: evolution by horizontal gene transfer and independent domain fusion. Mol Biol Evol. 2014:31(2):376–386. 10.1093/molbev/mst206 24150040

[koaf195-B82] Ocaña-Pallarès E, Najle SR, Scazzocchio C, Ruiz-Trillo I. Reticulate evolution in eukaryotes: origin and evolution of the nitrate assimilation pathway. PLoS Genet. 2019:15(2):e1007986. 10.1371/journal.pgen.1007986 30789903 PMC6400420

[koaf195-B83] Ocaña-Pallarès E, Williams TA, López-Escardó D, Arroyo AS, Pathmanathan JS, Bapteste E, Tikhonenkov DV, Keeling PJ, Szöllősi GJ, Ruiz-Trillo I. Divergent genomic trajectories predate the origin of animals and fungi. Nature. 2022:609(7928):747–753. 10.1038/s41586-022-05110-4 36002568 PMC9492541

[koaf195-B84] Ott JP, Klimešová J, Hartnett DC. The ecology and significance of below-ground bud banks in plants. Ann Bot. 2019:123(7):1099–1118. 10.1093/aob/mcz051 31167028 PMC6612937

[koaf195-B85] Paajanen P, Tomkins M, Hoerbst F, Veevers R, Heeney M, Thomas HR, Apelt F, Saplaoura E, Gupta S, Frank M, et al Re-analysis of mobile mRNA datasets raises questions about the extent of long-distance mRNA communication. Nat Plants. 2025:11(5):977–984. 10.1038/s41477-025-01979-x 40240650 PMC12095074

[koaf195-B86] Panchy N, Lehti-Shiu M, Shiu S-H. Evolution of gene duplication in plants. Plant Physiol. 2016:171(4):2294–2316. 10.1104/pp.16.00523 27288366 PMC4972278

[koaf195-B87] Park M, Christin P-A, Bennetzen JL. Sample sequence analysis uncovers recurrent horizontal transfers of transposable elements among grasses. Mol Biol Evol. 2021:38(9):3664–3675. 10.1093/molbev/msab133 33964159 PMC8382918

[koaf195-B88] Peccoud J, Cordaux R, Gilbert C. Analyzing horizontal transfer of transposable elements on a large scale: challenges and prospects. BioEssays. 2018:40(2):1700177. 10.1002/bies.201700177 29283188

[koaf195-B89] Peng H, Mirouze M, Bucher E. Extrachromosomal circular DNA: a neglected nucleic acid molecule in plants. Curr Opin Plant Biol. 2022:69:102263. 10.1016/j.pbi.2022.102263 35872391

[koaf195-B90] Pereira L, Christin P-A, Dunning LT. The mechanisms underpinning lateral gene transfer between grasses. Plants People Planet. 2023:5(5):672–682. 10.1002/ppp3.10347

[koaf195-B91] Pereira L, Dunning LT. Extrachromosomal circular DNA as a vehicle to gene transfer in plants. Plant Physiol. 2023:193(1):172–173. 10.1093/plphys/kiad380 37394915 PMC10469358

[koaf195-B92] Peumans WJ, Fouquaert E, Jauneau A, Rougé P, Lannoo N, Hamada H, Alvarez R, Devreese B, Van Damme EJM. The liverwort *Marchantia polymorpha* expresses orthologs of the fungal *Agaricus bisporus* agglutinin family. Plant Physiol. 2007:144(2):637–647. 10.1104/pp.106.087437 17041032 PMC1914199

[koaf195-B93] Puginier C, Libourel C, Otte J, Skaloud P, Haon M, Grisel S, Petersen M, Berrin J-G, Delaux P-M, Dal Grande F, et al Phylogenomics reveals the evolutionary origins of lichenization in chlorophyte algae. Nat Commun. 2024:15(1):4452. 10.1038/s41467-024-48787-z 38789482 PMC11126685

[koaf195-B94] Qu J, Xu S, Zhang Z, Chen G, Zhong Y, Liu L, Zhang R, Xue J, Guo D. Evolutionary, structural and expression analysis of core genes involved in starch synthesis. Sci Rep. 2018:8(1):12736. 10.1038/s41598-018-30411-y 30143668 PMC6109180

[koaf195-B95] Quispe-Huamanquispe DG, Gheysen G, Kreuze JF. Horizontal gene transfer contributes to plant evolution: the case of Agrobacterium T-DNAs. Front Plant Sci. 2017:8:2015. 10.3389/fpls.2017.02015 29225610 PMC5705623

[koaf195-B96] Quispe-Huamanquispe DG, Gheysen G, Yang J, Jarret R, Rossel G, Kreuze JF. The horizontal gene transfer of Agrobacterium T-DNAs into the series Batatas (Genus Ipomoea) genome is not confined to hexaploid sweetpotato. Sci Rep. 2019:9(1):12584. 10.1038/s41598-019-48691-3 31467320 PMC6715720

[koaf195-B97] Raimondeau P, Bianconi ME, Pereira L, Parisod C, Christin P-A, Dunning LT. Lateral gene transfer generates accessory genes that accumulate at different rates within a grass lineage. New Phytol. 2023:240(5):2072–2084. 10.1111/nph.19272 37793435

[koaf195-B98] Reape TJ, McCabe PF. Apoptotic-like programmed cell death in plants. New Phytol. 2008:180(1):13–26. 10.1111/j.1469-8137.2008.02549.x 18631291

[koaf195-B99] Remigi P, Capela D, Clerissi C, Tasse L, Torchet R, Bouchez O, Batut J, Cruveiller S, Rocha EPC, Masson-Boivin C. Transient hypermutagenesis accelerates the evolution of legume endosymbionts following horizontal gene transfer. PLoS Biol. 2014:12(9):e1001942. 10.1371/journal.pbio.1001942 25181317 PMC4151985

[koaf195-B100] Rensing SA . Great moments in evolution: the conquest of land by plants. Curr Opin Plant Biol. 2018:42:49–54. 10.1016/j.pbi.2018.02.006 29525128

[koaf195-B101] Richards TA, Soanes DM, Foster PG, Leonard G, Thornton CR, Talbot NJ. Phylogenomic analysis demonstrates a pattern of rare and ancient horizontal gene transfer between plants and fungi. Plant Cell. 2009:21(7):1897–1911. 10.1105/tpc.109.065805 19584142 PMC2729602

[koaf195-B102] Richardson AO, Palmer JD. Horizontal gene transfer in plants. J Exp Bot. 2007:58(1):1–9. 10.1093/jxb/erl148 17030541

[koaf195-B103] Sanchez-Puerta MV, García LE, Wohlfeiler J, Ceriotti LF. Unparalleled replacement of native mitochondrial genes by foreign homologs in a holoparasitic plant. New Phytol. 2017:214(1):376–387. 10.1111/nph.14361 27905116

[koaf195-B104] Sanders WB, Masumoto H. Lichen algae: the photosynthetic partners in lichen symbioses. Lichenologist. 2021:53(5):347–393. 10.1017/S0024282921000335

[koaf195-B105] Sevillya G, Adato O, Snir S. Detecting horizontal gene transfer: a probabilistic approach. BMC Genomics. 2020:21(S1):106. 10.1186/s12864-019-6395-5 32138652 PMC7057450

[koaf195-B106] Shimodaira H . An approximately unbiased test of phylogenetic tree selection. Syst Biol. 2002:51(3):492–508. 10.1080/10635150290069913 12079646

[koaf195-B107] Shinozuka H, Shinozuka M, de Vries EM, Sawbridge TI, Spangenberg GC, Cocks BG. Fungus-originated genes in the genomes of cereal and pasture grasses acquired through ancient lateral transfer. Sci Rep. 2020:10(1):19883. 10.1038/s41598-020-76478-4 33199756 PMC7670438

[koaf195-B108] Shukla AK, Upadhyay SK, Mishra M, Saurabh S, Singh R, Singh H, Thakur N, Rai P, Pandey P, Hans AL, et al Expression of an insecticidal fern protein in cotton protects against whitefly. Nat Biotechnol. 2016:34(10):1046–1051. 10.1038/nbt.3665 27598229

[koaf195-B109] Spribille T, Resl P, Stanton DE, Tagirdzhanova G. Evolutionary biology of lichen symbioses. New Phytol. 2022:234(5):1566–1582. 10.1111/nph.18048 35302240

[koaf195-B110] Steenwyk JL, Li Y, Zhou X, Shen X-X, Rokas A. Incongruence in the phylogenomics era. Nat Rev Genet. 2023:24(12):834–850. 10.1038/s41576-023-00620-x 37369847 PMC11499941

[koaf195-B111] Stegemann S, Bock R. Exchange of genetic material between cells in plant tissue grafts. Science. 2009:324(5927):649–651. 10.1126/science.1170397 19407205

[koaf195-B112] Stegemann S, Keuthe M, Greiner S, Bock R. Horizontal transfer of chloroplast genomes between plant species. Proc Natl Acad Sci U S A. 2012:109(7):2434–2438. 10.1073/pnas.1114076109 22308367 PMC3289295

[koaf195-B113] Stolzer M, Lai H, Xu M, Sathaye D, Vernot B, Durand D. Inferring duplications, losses, transfers and incomplete lineage sorting with nonbinary species trees. Bioinformatics. 2012:28(18):i409–i415. 10.1093/bioinformatics/bts386 22962460 PMC3436813

[koaf195-B114] Takizawa R, Hatada M, Moriwaki Y, Abe S, Yamashita Y, Arimitsu R, Yamato KT, Nishihama R, Kohchi T, Koeduka T, et al Fungal-type terpene synthases in *Marchantia polymorpha* are involved in sesquiterpene biosynthesis in oil body cells. Plant Cell Physiol. 2021:62(3):528–537. 10.1093/pcp/pcaa175 33439267

[koaf195-B115] Tan S, Cardoso-Moreira M, Shi W, Zhang D, Huang J, Mao Y, Jia H, Zhang Y, Chen C, Shao Y, et al LTR-mediated retroposition as a mechanism of RNA-based duplication in metazoans. Genome Res. 2016:26(12):1663–1675. 10.1101/gr.204925.116 27934698 PMC5131818

[koaf195-B116] Tarrío R, Ayala FJ, Rodríguez-Trelles F. The vein patterning 1 (VEP1) gene family laterally spread through an ecological network. PLoS One. 2011:6(7):e22279. 10.1371/journal.pone.0022279 21818306 PMC3144213

[koaf195-B117] Thieme CJ, Rojas-Triana M, Stecyk E, Schudoma C, Zhang W, Yang L, Miñambres M, Walther D, Schulze WX, Paz-Ares J, et al Endogenous Arabidopsis messenger RNAs transported to distant tissues. Nat Plants. 2015:1(4):1–9. 10.1038/nplants.2015.25 27247031

[koaf195-B118] Van Hautegem T, Waters AJ, Goodrich J, Nowack MK. Only in dying, life: programmed cell death during plant development. Trends Plant Sci. 2015:20(2):102–113. 10.1016/j.tplants.2014.10.003 25457111

[koaf195-B119] Wang H, Li Y, Zhang Z, Zhong B. Horizontal gene transfer: driving the evolution and adaptation of plants. J Integr Plant Biol. 2023:65(3):613–616. 10.1111/jipb.13407 36354153

[koaf195-B120] Wang H, Sun S, Ge W, Zhao L, Hou B, Wang K, Lyu Z, Chen L, Xu S, Guo J, et al Horizontal gene transfer of *Fhb7* from fungus underlies *Fusarium* head blight resistance in wheat. Science. 2020a:368(6493):eaba5435. 10.1126/science.aba5435 32273397

[koaf195-B121] Wang K, Guo G, Bai S, Ma J, Zhang Z, Xing Z, Wang W, Li H, Liang H, Li Z, et al Horizontally acquired CSP genes contribute to wheat adaptation and improvement. Nat Plants. 2025:11(4):761–774. 10.1038/s41477-025-01952-8 40148598

[koaf195-B122] Wang L, Xing H, Yuan Y, Wang X, Saeed M, Tao J, Feng W, Zhang G, Song X, Sun X. Genome-wide analysis of codon usage bias in four sequenced cotton species. PLoS One. 2018:13(3):e0194372. 10.1371/journal.pone.0194372 29584741 PMC5870960

[koaf195-B123] Wang Y-W, Hess J, Slot JC, Pringle A. De Novo gene birth, horizontal gene transfer, and gene duplication as sources of new gene families associated with the origin of symbiosis in *Amanita*. Genome Biol Evol. 2020b:12(11):2168–2182. 10.1093/gbe/evaa193 32926145 PMC7674699

[koaf195-B124] Waseem M, Nkurikiyimfura O, Niyitanga S, Jakada BH, Shaheen I, Aslam MM. GRAS transcription factors emerging regulator in plants growth, development, and multiple stresses. Mol Biol Rep. 2022:49(10):9673–9685. 10.1007/s11033-022-07425-x 35713799

[koaf195-B125] Watanabe Y, Spangenberg GC, Shinozuka H. Fungus-originated glucanase and monooxygenase genes in creeping bent grass (Agrostis stolonifera L.). PLoS One. 2021:16(9):e0257173. 10.1371/journal.pone.0257173 34506557 PMC8432771

[koaf195-B126] Wickell DA, Li F-W. On the evolutionary significance of horizontal gene transfers in plants. New Phytol. 2020:225(1):113–117. 10.1111/nph.16022 31347197

[koaf195-B127] Won H, Renner SS. Horizontal gene transfer from flowering plants to Gnetum. Proc Natl Acad Sci U S A. 2003:100(19):10824–10829. 10.1073/pnas.1833775100 12963817 PMC196887

[koaf195-B128] Wu S, Jandrasits K, Swarts K, Roetzer J, Akimcheva S, Shimamura M, Hisanaga T, Berger F, Dolan L. Population genomics of *Marchantia polymorpha* subsp. *ruderalis* reveals evidence of climate adaptation. Curr Biol. 2025:35(5):970–980.e3. 10.1016/j.cub.2025.01.008 39933518

[koaf195-B129] Xi Z, Bradley RK, Wurdack KJ, Wong K, Sugumaran M, Bomblies K, Rest JS, Davis CC. Horizontal transfer of expressed genes in a parasitic flowering plant. BMC Genomics. 2012:13(1):227. 10.1186/1471-2164-13-227 22681756 PMC3460754

[koaf195-B130] Xia J, Guo Z, Yang Z, Han H, Wang S, Xu H, Yang X, Yang F, Wu Q, Xie W, et al Whitefly hijacks a plant detoxification gene that neutralizes plant toxins. Cell. 2021:184(7):1693–1705.e17. 10.1016/j.cell.2021.02.014 33770502

[koaf195-B131] Yadav SK, Archana, Singh R, Singh PK, Vasudev PG. Insecticidal fern protein Tma12 is possibly a lytic polysaccharide monooxygenase. Planta. 2019:249(6):1987–1996. 10.1007/s00425-019-03135-0 30903269

[koaf195-B132] Yang Z, Wafula EK, Kim G, Shahid S, McNeal JR, Ralph PE, Timilsena PR, Yu W, Kelly EA, Zhang H, et al Convergent horizontal gene transfer and cross-talk of mobile nucleic acids in parasitic plants. Nat Plants. 2019:5(9):991–1001. 10.1038/s41477-019-0458-0 31332314

[koaf195-B133] Yang Z, Zhang Y, Wafula EK, Honaas LA, Ralph PE, Jones S, Clarke CR, Liu S, Su C, Zhang H, et al Horizontal gene transfer is more frequent with increased heterotrophy and contributes to parasite adaptation. Proc Nat Acad Sci U S A. 2016:113(45):E7010–E7019. 10.1073/pnas.1608765113 PMC5111717 27791104

[koaf195-B134] Yang Z, Zhou Y, Huang J, Hu Y, Zhang E, Xie Z, Ma S, Gao Y, Song S, Xu C, et al Ancient horizontal transfer of transaldolase-like protein gene and its role in plant vascular development. New Phytol. 2015:206(2):807–816. 10.1111/nph.13183 25420550 PMC4407918

[koaf195-B135] Yoshida S, Maruyama S, Nozaki H, Shirasu K. Horizontal gene transfer by the parasitic plant *Striga hermonthica*. Science. 2010:328(5982):1128. 10.1126/science.1187145 20508124

[koaf195-B136] Yu L, Boström C, Franzenburg S, Bayer T, Dagan T, Reusch TBH. Somatic genetic drift and multilevel selection in a clonal seagrass. Nat Ecol Evol. 2020:4(7):952–962. 10.1038/s41559-020-1196-4 32393866

[koaf195-B137] Yue J, Hu X, Sun H, Yang Y, Huang J. Widespread impact of horizontal gene transfer on plant colonization of land. Nat Commun. 2012:3(1):1152. 10.1038/ncomms2148 23093189 PMC3493653

[koaf195-B138] Zhang D, Qi J, Yue J, Huang J, Sun T, Li S, Wen J-F, Hettenhausen C, Wu J, Wang L, et al Root parasitic plant Orobanche aegyptiaca and shoot parasitic plant Cuscuta australis obtained Brassicaceae-specific strictosidine synthase-like genes by horizontal gene transfer. BMC Plant Biol. 2014:14(1):19. 10.1186/1471-2229-14-19 24411025 PMC3893544

[koaf195-B139] Zhang P, Mbodj A, Soundiramourtty A, Llauro C, Ghesquière A, Ingouff M, Keith Slotkin R, Pontvianne F, Catoni M, Mirouze M. Extrachromosomal circular DNA and structural variants highlight genome instability in Arabidopsis epigenetic mutants. Nat Commun. 2023:14(1):5236. 10.1038/s41467-023-41023-0 37640706 PMC10462705

[koaf195-B140] Zhang Y, Fernandez-Aparicio M, Wafula EK, Das M, Jiao Y, Wickett NJ, Honaas LA, Ralph PE, Wojciechowski MF, Timko MP, et al Evolution of a horizontally acquired legume gene, albumin 1, in the parasitic plant phelipanche aegyptiaca and related species. BMC Evol Biol. 2013:13(1):48. 10.1186/1471-2148-13-48 23425243 PMC3601976

[koaf195-B141] Zhu Q, Kosoy M, Dittmar K. HGTector: an automated method facilitating genome-wide discovery of putative horizontal gene transfers. BMC Genomics. 2014:15(1):717. 10.1186/1471-2164-15-717 25159222 PMC4155097

[koaf195-B142] Zhu Z, Tan S, Zhang Y, Zhang YE. LINE-1-like retrotransposons contribute to RNA-based gene duplication in dicots. Sci Rep. 2016:6(1):24755. 10.1038/srep24755 27098918 PMC4838847

[koaf195-B143] Zhuang J, Zhang Y, Zhou C, Fan D, Huang T, Feng Q, Lu Y, Zhao Y, Zhao Q, Han B, et al Dynamics of extrachromosomal circular DNA in rice. Nat Commun. 2024:15(1):2413. 10.1038/s41467-024-46691-0 38499575 PMC10948907

